# What are the demographic predictors and barriers to self-care engagement in the UK? a cross-sectional survey study

**DOI:** 10.1136/bmjopen-2025-110380

**Published:** 2026-09-21

**Authors:** Austen El-Osta, Sami Altalib, Mahmoud Al Ammouri, Peter Smith

**Affiliations:** 1 Department of Primary Care and Public Health, Imperial College London, London, UK; 2 The Self-Care Forum UK, Surrey, UK

**Keywords:** PUBLIC HEALTH, Primary Health Care, Health policy

## Abstract

**Abstract:**

**Background:**

Understanding how people manage everyday health challenges is vital for developing self-care policies that are inclusive, equitable and effective. Little is known about how demographic characteristics shape self-care confidence, symptom management strategies and health-seeking behaviours across the UK adult population. While the companion paper from the same survey examined healthcare professionals’ attitudes toward self-care and the professional-public interface, the demographic predictors and personal barriers shaping self-care engagement among the general UK public remain poorly understood.

**Objective:**

To examine the demographic predictors of self-care engagement among UK adults, focusing on self-care confidence, health information-seeking behaviour, symptom management strategies and perceived barriers to self-care.

**Design:**

Cross-sectional online survey conducted from June to September 2024.

**Setting:**

Community-dwelling adults across the UK, recruited via online platforms and professional networks.

**Participants:**

3,255 UK adults, including a subset of health and care professionals (HCPs).

**Interventions:**

None (observational study).

**Primary and secondary outcome measures:**

Self-reported self-care confidence, health information-seeking behaviours, symptom management strategies and barriers to self-care.

**Results:**

Regression analyses highlighted marked demographic disparities in self-care engagement. Older adults (65+) were significantly more confident in their self-care knowledge (aOR=3.22, 95% CI 2.06 to 5.03, p<0.001) and healthy lifestyle behaviours (aOR=2.96) and were also more likely to seek health information. Males reported lower self-care confidence than females (aOR=0.79, 95% CI 0.68 to 0.91, p=0.002). Black British participants were more confident in self-care knowledge (aOR=1.99, 95% CI 1.39 to 2.84, p<0.001), but along with Asian British individuals, were significantly less likely to seek health information (aORs=0.56 and 0.70, respectively). People living with disabilities (aOR=0.69) and long-term conditions (aOR=0.76) reported lower confidence across self-care domains. HCPs consistently reported higher self-care confidence (aOR=1.51, p=0.006) and health behaviour engagement (aOR=1.66). Financial constraints (53%), lack of time (47%) and low self-efficacy (22%) emerged as key barriers to self-care, alongside low use of pharmacists (1.3%) and digital resources (9.7%).

**Conclusion:**

This study highlights demographic disparities in self-care confidence, information-seeking behaviours and barriers to engagement, urging the need for tailored self-care interventions. Future research should focus on targeted interventions to improve health literacy, enhance pharmacist-led self-care support and promote equitable access to digital health resources, particularly for underserved and demographically diverse population groups.

**STRENGTHS AND LIMITATIONS OF THIS STUDY**

- A large convenience sample of 3255 UK adults, recruited across all four UK nations, provides demographic breadth within the digitally connected adult population.
- The sample was recruited by open, non-probability methods and is not representative of the UK adult population; adults aged 65 and over are under-represented and graduates over-represented, so findings describe associations within the sample rather than national prevalence.
- Application of ordinal logistic regression adjusted for multiple demographic confounders allowed robust identification of predictors of self-care engagement.
- Online recruitment may have introduced selection bias by excluding individuals with limited internet access or low digital literacy, potentially underrepresenting certain vulnerable groups.
- Cross-sectional design limits causal inference and relies on self-reported data, which may be subject to recall and social desirability biases.

## Introduction

 There are over 130 definitions of self-care in the academic literature,^1^ including five definitions from the WHO. In simple terms, self-care is our ability to maintain our health and well-being and to prevent, delay or change the trajectory of common lifestyle diseases.^2^ It is increasingly recognised as a cornerstone of sustainable health and care systems, particularly in light of growing health and care demands, workforce shortages and the rising burden of chronic diseases.^3 4^ Effective self-care practices can reduce pressure on health and care services and empower individuals to take greater responsibility for their health and well-being.^5 6^ Despite these recognised benefits, self-care engagement is neither uniform nor equitable across populations, with disparities in self-care confidence, health literacy, access to information and encouragement from health and care professionals (HCPs) contributing to variable adoption and effectiveness of self-care strategies.^7–9^

Understanding how individuals engage with and navigate self-care practices is crucial for designing targeted public health interventions that enhance self-care literacy, improve health outcomes and support health and care system sustainability. Previous research, including the Living Self-Care Survey (LSCS) Study, examined the public–HCP interface, revealing significant demographic disparities in self-care confidence, health-seeking behaviours and engagement with professional guidance.^8–10^ Self-care confidence, as operationalised here, refers to a global self-appraisal of one’s knowledge and ability to manage common and self-limiting health conditions independently. In this conception, it is broader in scope than domain-specific self-efficacy and distinct from patient activation and health literacy, which focus on healthcare engagement and information appraisal, respectively.

Despite increasing policy emphasis on self-care,^3^ there remains a limited empirical understanding of how demographic differences influence self-care behaviours and health-seeking practices. One critical gap relates to self-care confidence, which varies across age, gender and ethnicity, yet the underlying factors shaping these differences remain poorly understood.^11–13^ Evidence on age and self-care confidence is mixed: some studies report lower confidence among older adults alongside greater health information-seeking, suggesting potential barriers to translating knowledge into effective self-care actions particularly for people living with multiple long-term conditions.^14^ Conversely, some ethnic groups exhibit higher confidence but lower engagement with formal health resources,^15 16^ raising questions about trust in health and care systems, cultural perceptions of self-care and alternative health-seeking behaviours.

Another pressing issue is health literacy and information-seeking behaviours, which are fundamental to effective self-care but remain highly variable across demographic groups.^17–19^ The increasing reliance on digital health resources as a means of promoting self-care has created new challenges related to accessibility, digital literacy and the credibility of online health information. Individuals with lower health literacy or limited digital access may struggle to interpret and apply self-care guidance effectively, leading to misinformed decision-making, increased dependency on health and care services or disengagement from self-care practices altogether.^20 21^ Understanding how different population groups access, evaluate and trust health information is critical in the context of the ‘symptom iceberg’, alluding to the silent majority of symptoms managed outside formal health and care settings.

The companion analysis of this survey^8^ examined the public–professional interface: how HCP view self-care, the barriers they perceive on behalf of their patients and population-level health literacy. It did not model the general public’s own self-care engagement, and three questions therefore remain open. First, which demographic characteristics independently predict self-care confidence and health information-seeking among the public once other characteristics are held constant? Second, which personal and structural barriers does the public itself identify, as distinct from those attributed to it by professionals? Third, how do symptom management strategies and use of professional resources vary across specific common conditions? Addressing these in a single sample of 3255 adults provides the first large-scale, demographically stratified and symptom-level account of public self-care behaviour in the UK and permits direct comparison with Elliott *et al*’s 2011 characterisation of the symptom iceberg^22^: reliance on over-the-counter medicines for common symptoms has approximately doubled over the intervening decade while consultation with any health or care professional has more than halved, with community pharmacy—the access point most heavily promoted by national self-care policy over that period—used in fewer than one in seventy symptom episodes.

## Methods

### Study design and data collection

This study is part of the LSCS Study, a cross-sectional, UK-wide online survey investigating self-care confidence, behaviours and health information-seeking practices. The survey was conducted between 1 June and 30 September 2024 (3 months) using the Imperial Qualtrics XM online platform. Participants were recruited through an open, non-probability approach. The survey link was posted publicly on the social media accounts of the Self-Care Academic Research Unit and the Self-Care Forum (X and LinkedIn) and circulated through the mailing lists, newsletters and professional networks of partner health and care organisations. The link was accessible to anyone who saw the post or received the circular; it was not sent to individual inboxes and the research team held no personal contact details for any participant. There was therefore no closed sampling frame and no denominator from which a conventional response rate could be calculated. The survey consisted of 83 questions displayed across 15 screens, structured into 6 thematic blocks covering: demographic characteristics, health and well-being, self-care knowledge and practices, barriers to self-care, symptom management and HCP-specific questions. Adaptive questioning was employed so that only relevant items were displayed: symptom-management items appeared only for symptoms the respondent reported experiencing, long-term condition items only for those reporting such a condition and the professional block only for those identifying as health or care professionals. No respondent was therefore shown all 83 items. To assess whether the length of the instrument induced satisficing, we quantified straightlining within the two longest Likert blocks. In the eight-item attitudes block, which contains both positively and negatively framed statements, 0.9% of respondents selected an identical response to every item and in the 13-item health literacy block the corresponding figure was 4.8%. The survey was piloted with 21 participants (6 departmental colleagues and 15 members of the public representing diverse demographics), with brief personal interviews conducted to assess comprehension; modifications were made accordingly. To ensure data quality, browser cookies and IP address checks prevented duplicate submissions, and responses with unrealistic completion times were excluded. All survey data were stored on the Imperial College London securely encrypted online research data portal, accessible only to the research team following login with credentials. Responses were pseudo-anonymised by assigning each respondent a unique study ID; only demographic data (age, gender, ethnicity, employment type, highest education level and region of residence) were recorded. Respondents could decline to answer any item by selecting 'prefer not to say’. All reported health conditions, disabilities and symptoms are self-reported and have not been clinically verified.

### Study participants

The study sample consists of 3255 community-dwelling UK adults, including a subset of HCPs, who completed the LSCS survey. Adults aged ≥18 years, residing in the UK, with internet access and the ability to complete an online survey in English were eligible. Incomplete survey responses and duplicate entries were excluded from analysis. The sample is a self-selected convenience sample of digitally connected UK adults and is not a probability sample of the UK adult population. The descriptive percentages reported below therefore describe this sample and should not be interpreted as national prevalence estimates, and the regression models estimate associations within the sample rather than population-level parameters.

### Measures and variables

This study examines self-care engagement through five key domains: (i) demographic predictors of self-care knowledge, confidence and information accessibility (age, gender, ethnicity, education and HCP status), (ii) confidence in leading a healthy lifestyle, including engagement with preventive health behaviours, (iii) health-seeking behaviours and symptom self-management for common conditions, (iv) personal barriers to self-care, such as financial constraints, time limitations and lack of support and (v) influence of HCP encouragement on self-care adoption across demographic subgroups. Each domain was operationalised using items from the survey instrument, which is reproduced in full in the supplementary material of the companion paper.^8^ The 13 health literacy items were adapted from the European Health Literacy Survey Questionnaire,^23^ and the self-care confidence, attitude, activity and barrier items were drawn from the Self-Care Forum’s LSCS instrument.^8 9^

Self-care confidence (domains i and ii) was measured using three 4-point forced-choice ordinal items with no neutral midpoint ("How confident are you that you have the knowledge and understanding to: lead a healthy lifestyle? / practise self-care? / manage common illnesses?"; response options: not at all confident, not very confident, fairly confident, very confident). Health information-seeking behaviour (domain iii) was assessed using a 5-point agreement item (“When I have a common condition, I try to find health information for myself”; strongly disagree to strongly agree). Symptom management strategies (domain iii) were captured using multiple-response tick-box items for each of the 10 specified common symptoms. Personal barriers (domain iv) were measured via a multiple-response checklist of predefined barrier categories.

The three confidence items showed acceptable internal consistency (Cronbach’s alpha 0.78; mean inter-item correlation 0.55; corrected item-total correlations 0.54–0.67; n=3255). As the items are ordinal, we also report ordinal alpha computed from the polychoric correlation matrix (0.86). Internal consistency for the health literacy block was good (Cronbach’s alpha 0.88). These items were analysed as separate single-item outcomes rather than as a summed composite, and these statistics are therefore reported as evidence that the items behave coherently rather than as psychometric validation. Notwithstanding this acceptable internal consistency, the confidence items have not undergone full psychometric validation and no assessment of factor structure, measurement invariance across demographic groups or test–retest stability has been conducted. They reflect subjective self-appraisal and may carry different meanings across demographic and cultural groups. Therefore, findings should be interpreted descriptively rather than as definitive measures of self-care capacity.

### Statistical analysis

No a priori sample size calculation was conducted; the achieved sample was determined pragmatically by the recruitment window. While sufficient for main-effect ordinal regression, analyses for smaller subgroups (eg, specific ethnic minority groups) may have limited power and should be interpreted with caution. Survey responses were analysed using descriptive statistics and ordinal logistic regression models to identify demographic predictors of self-care engagement. Participants selecting 'Prefer not to say’ or leaving items blank were excluded on a complete-case basis (listwise deletion). Missing data on individual covariates ranged from 0.4% (education) to 4.1% (long-term condition status); listwise deletion yielded an analytic sample of 3011 (92.5%), and given this modest loss imputation was not performed. Adjusted regression models controlled for age, gender, ethnicity, education, employment status, disability, long-term conditions, HCP status and country of residence. Covariates were specified a priori on conceptual and literature grounds rather than by any statistical criterion, each being an established determinant of health literacy, health-seeking behaviour or self-care capability^7–9 14–19^ and plausibly associated with both the demographic exposures and the outcomes. No stepwise, forward or backward selection procedure was used, no variable was added to or removed from the models on the basis of its p value, and the same fully adjusted model was fitted for every outcome. HCP status was included as a comparator covariate to contextualise professional versus lay self-care behaviours, consistent with the analytical approach of the companion study,^8^ rather than as a primary demographic predictor. The results were reported using OR and adjusted odds ratios (aOR) with 95% CIs. Statistical significance was set at p<0.05. The proportional odds assumption was assessed using the Brant test, evaluated globally and separately for each covariate. Because both the lowest outcome categories (28–50 respondents across the confidence outcomes, 11 for information-seeking) and the lowest education category (n=7) were sparsely populated, the test was not estimable on the full specifications; these sparse categories were combined for the purposes of the assumption test only, yielding estimates that differ from those reported by less than 0.05 on the OR scale. The global test indicated departure from proportionality in all three models (self-care knowledge χ²=53.31, df=26, p=0.001; healthy lifestyle χ²=39.31, df=26, p=0.046; information-seeking χ²=62.71, df=26, p<0.001). Covariate-specific testing localised these departures to five, three and five covariates respectively, with all remaining covariates consistent with proportional odds. For flagged covariates that were also significant predictors, threshold-specific estimates preserved the direction of the pooled estimate at every threshold except in two cases, and preserved statistical significance at both thresholds except in two further instances. The directional exceptions were Black British ethnicity in the self-care knowledge model, whose association is concentrated at the upper end of the confidence distribution and Northern Ireland residency in the healthy lifestyle model, estimated on 69 respondents. Gender and postgraduate education in the self-care knowledge model retained their direction but attenuated to non-significance at the highest confidence threshold. The ordinal specification was therefore retained, with explicit caveats on these two estimates. With 27 covariates in the reported models and an analytic sample of 3,011, the omnibus test is highly powered and will detect departures too small to alter any inference.

Multicollinearity was assessed using generalised variance inflation factors (GVIF) for multi-category predictors and variance inflation factors with tolerance for individual indicators. All GVIF^∧^(1/2×df) values lay between 1.01 and 1.13, indicating no meaningful collinearity between predictor sets. Indicator-level variance inflation factors for the education dummies were high (48.8–109.5), but this reflects the very small primary school reference category (n=7) rather than dependency between predictors: the generalised factor for education, which is invariant to the choice of reference category, was 1.02. All other indicator-level factors were ≤3.48 with a minimum tolerance of 0.29. No predictor was removed or combined on collinearity grounds, and the education estimates are interpreted with the caution noted below.

Overall model adequacy was assessed using the likelihood ratio test against the intercept-only model, McFadden, Cox–Snell and Nagelkerke pseudo-R² and the Akaike and Bayesian information criteria. These models were specified to estimate adjusted associations rather than to predict individual-level behaviour, and the pseudo-R² values are reported as descriptive indices of fit rather than as measures of predictive performance. Reference categories for all categorical variables were selected based on the largest group in the sample or the most widely used comparator in the UK health disparities literature, to maximise statistical stability and ensure comparability with existing evidence. Specifically: age 18–24; female gender; White British ethnicity (the largest group in the 2021 UK Census); no disability; no long-term condition; non-HCP and unemployed. For education, primary school was used as the lowest attainment category. Education-level associations should be interpreted with caution, given the very small reference category, which yields unstable estimates and wide CIs.

Because recruitment was open and non-probability, no design weights exist, and selection probabilities are unknown. We considered post-stratification and raking to UK adult population margins and elected not to apply weights: the estimands are conditional associations rather than prevalences; age, gender, ethnicity, education and employment status are already covariates in every adjusted model, and weighting to observed demographic margins cannot correct selection on the unobserved characteristics likely to have driven participation, such as interest in self-care and digital access. Weighting would therefore increase variance without addressing the principal source of bias. All analyses were conducted using Stata V.17 (StataCorp LP, College Station, TX, US). The Checklist for Reporting Results of Internet E-Surveys was used to guide reporting.^24^

### Patient and Public involvement

The public was involved in the design of the study and questionnaire through piloting and feedback, contributed to data collection by distributing the survey and participated in disseminating the preliminary findings.

## Results

### Demographic profile of respondents

The survey included 3255 respondents, of whom the majority were aged between 25–34 years (23.0%) and 35–44 years (22.5%). The demographic characteristics of the study sample are itemised in online supplemental table S1.

### Prevalence of common symptoms and self-care responses

A significant proportion of respondents reported experiencing common symptoms in the 2 weeks prior to the survey, with headaches (49.5%), back pain (33.1%) and joint pain (27.1%) being the most frequently reported. Other common symptoms included sore throat (13.1%), indigestion/heartburn (17.9%), cough (14.0%) and cold or influenza symptoms (12.7%). Gastrointestinal symptoms such as diarrhoea (12.2%) and constipation (9.8%) were also noted, while COVID-19 symptoms were reported by 1.6% of respondents.

### Symptom management strategies

Most participants managed their symptoms independently, with self-care behaviours varying by symptom type. Headaches (71.9%), indigestion/heartburn (58.7%) and cold or influenza symptoms (56.6%) were primarily treated with over-the-counter (OTC) medications, while other conditions such as back pain (42.7%) and joint pain (53.0%) were more often left untreated, with individuals opting to wait for symptoms to resolve; online supplemental table S2. Reliance on HCPs was minimal across all symptoms, with 2.1% of respondents consulting a general practitioner (GP), except for COVID-19 symptoms (7.6%) and back pain (3.3%). Pharmacist consultations were less common at 1.3% for all symptoms.

### Patterns of self-care and health and care utilisation

The preference for OTC medication over seeking professional care suggests confidence in self-managing minor ailments. However, engagement with pharmacists (1.3%) and digital health resources (9.7%) was low, with only 3.1% checking the NHS website and 6.5% using the internet more generally. Less than one percent of respondents contacted NHS 111, suggesting that urgent care services were not a primary choice for symptom management.

Findings indicate a strong inclination toward self-management, with waiting and OTC medications being the predominant strategies. When faced with common minor ailments such as headaches, back pain or sore throats, the two most frequently adopted self-care strategies were taking OTC medications and waiting or doing nothing to see if symptoms improve. For instance, among those who experienced headaches (n=1610), 71.9% used OTC medicines and 25.0% chose to wait without seeking further advice or treatment. Similarly, for sore throats (n=427), 50.4% took OTC remedies and 43.6% opted to wait. These patterns were consistent across various symptoms, including indigestion (58.7% used OTC meds) and cold/influenza symptoms (56.6% used OTC meds, 37.1% waited). This high reliance on OTC products and passive monitoring highlights a cultural and behavioural tendency toward independent health management, especially for low-acuity conditions. It is further supported by self-reported data indicating that 46.5% ‘always’ and 43.5% ‘usually’ manage their health and common conditions themselves, with 56.3% agreeing and 37.9% strongly agreeing that they actively seek health information when experiencing symptoms. Importantly, confidence levels in managing health were high, with over 91% of participants reporting that they felt ‘fairly’ or ‘very’ confident in their knowledge and understanding to manage common illnesses. This self-efficacy was most pronounced in older adults (aOR=2.63, 95% CI 1.84 to 3.78, p<0.001 for ages 55–64), suggesting that age may enhance perceived competence in self-care.

### Personal barriers to self-care

The most frequently cited barrier was financial limitations, with 52.9% of respondents (n=1722) indicating that a lack of money prevented them from staying healthy or taking care of their common self-treatable or long-term health conditions. Similarly, time constraints were a major concern, cited by 46.7% of respondents (n=1520). A lack of confidence (22.3%) and insufficient support from HCPs (GPs/practice nurses: 20.1%; consultants/specialists: 16.4%) emerged as significant impediments. The co-occurrence of financial barriers and low self-care confidence raises a question of directionality. Financial constraints may erode confidence by limiting self-care opportunities; equally, low confidence may suppress engagement, widening socioeconomic disparities, indicating the need for future mediation or longitudinal analyses to disentangle these effects. Almost one in five (18.1%, n=589) cited a lack of knowledge about health and health and care issues, while 15.9% (n=519) reported difficulties accessing appropriate treatment information.

Almost one in five (19.2%, n=624) indicated that a lack of trustworthy information limited their ability to engage in self-care, whereas 8.2% (n=267) reported difficulty in understanding available health information, highlighting a potential gap in the accessibility and clarity of self-care resources. Other commonly reported barriers included a lack of home monitoring equipment (18.9%), a lack of training or skills (15.1%) and low personal interest in self-care (16.0%) indicating that motivational and educational interventions may be necessary to enhance engagement. Only a small proportion of respondents (5.4%) selected ‘Other’, while 1.5% (n=49) reported being unsure about what prevented them from engaging in self-care.

### Demographic predictors of self-care knowledge, confidence and information accessibility

**Figure 1:**
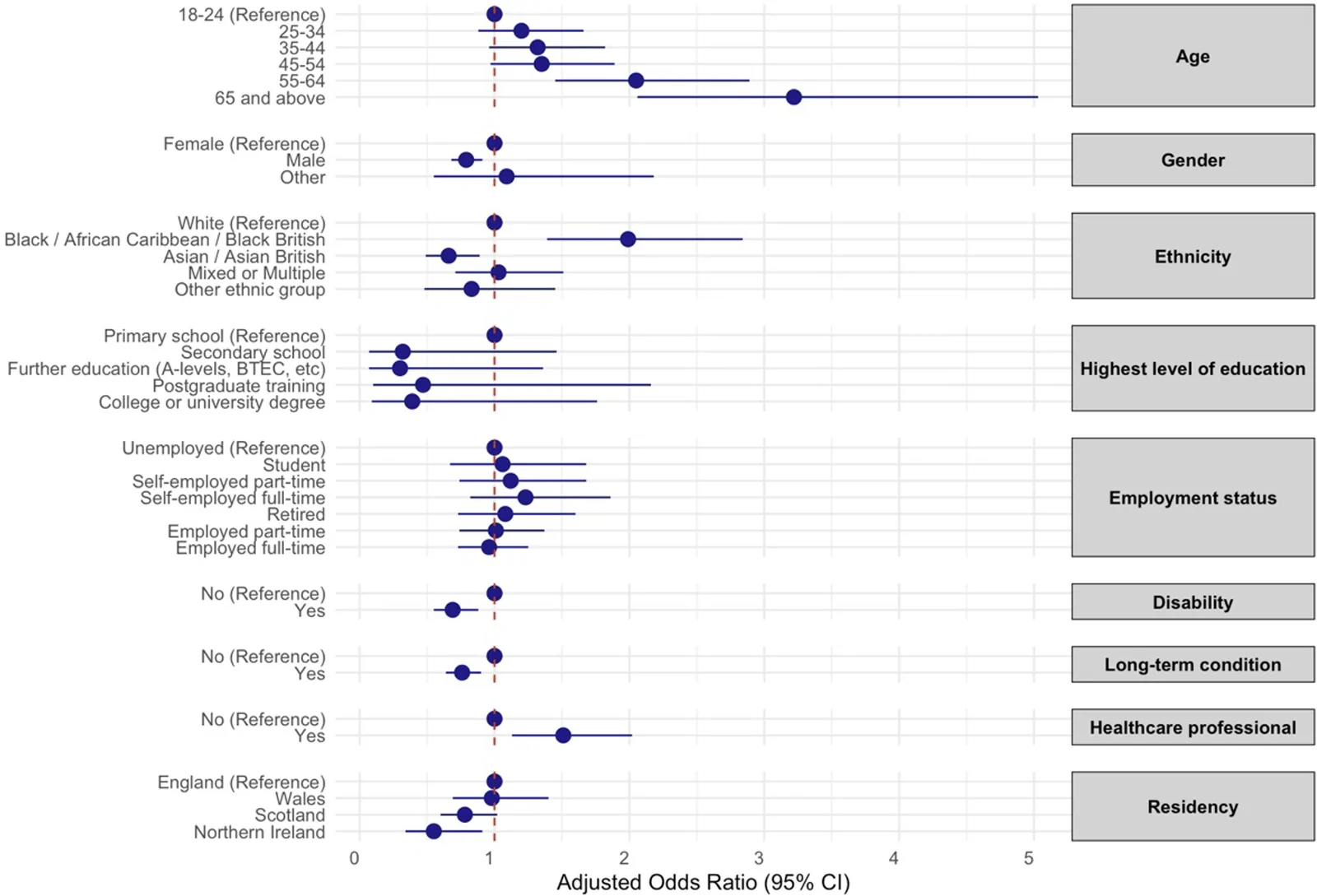
ORs and CIs - knowledge and understanding to practice self-care. Forest plot showing the ORs and CIs of factors associated with knowledge and understanding to practice self-care. BTEC, Business and Technology Education Council.

The ordinal logistic regression analysis presented in figure 1 **and** online supplemental table S3 identified significant demographic factors influencing confidence in knowledge and understanding of self-care practices. Respondents in the 55–64 age group had more than double the odds of reporting confidence in their self-care knowledge compared with the 18–24 reference group (aOR=2.05, 95% CI 1.45 to 2.89, p<0.001). Confidence increased further in the 65+ group (aOR=3.22, 95% CI 2.06 to 5.03, p<0.001). Males were less likely to report confidence in self-care compared with females (aOR=0.79, 95% CI 0.68 to 0.91, p=0.002). Ethnic differences were also significant; Black British individuals were more likely to report higher confidence than White participants (aOR=1.99, 95% CI 1.39 to 2.84, p<0.001), while Asian British individuals were less likely (aOR=0.66, 95% CI 0.49 to 0.89, p=0.006).

HCPs reported higher self-care confidence than non-professionals (aOR=1.51, 95% CI 1.13 to 2.02, p=0.006) highlighting the role of professional knowledge in self-care practices. Respondents in Northern Ireland reported lower confidence in their self-care knowledge than those in England (aOR=0.55, 95% CI 0.34 to 0.91, p=0.020), a pattern also observed for confidence in leading a healthy lifestyle (aOR=0.55, 95% CI 0.33 to 0.90, p=0.017), with no significant differences for Wales or Scotland. Confidence in information accessibility also varied: although a mean of 72% rated the health literacy items as easy or very easy, this ranged from 46% to 97% across items, with the lowest ratings for finding information on mental health problems and judging the advantages and disadvantages of treatment options.

### Confidence in leading a healthy lifestyle

**Figure 2:**
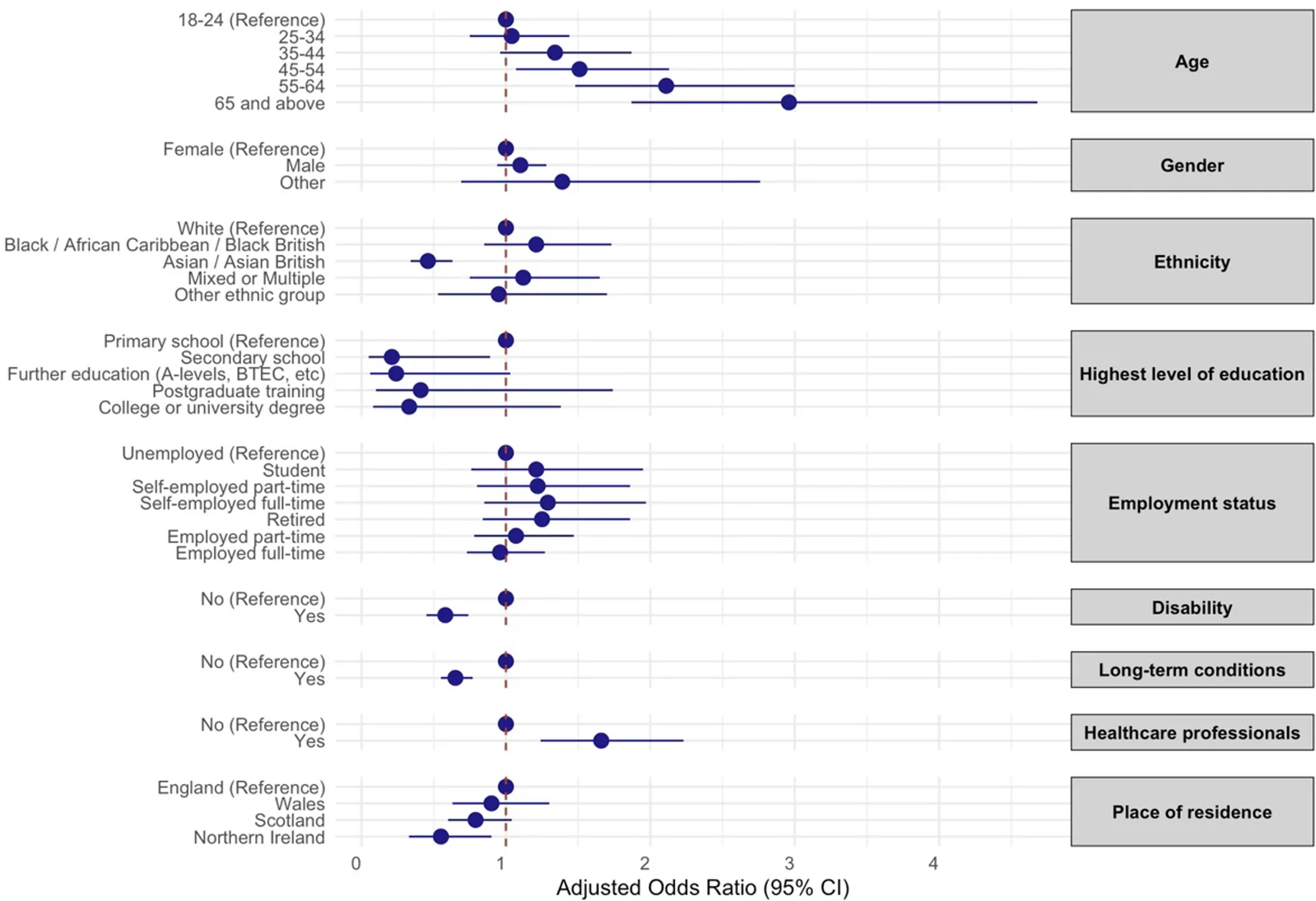
ORs and CIs - confidence in leading a healthy lifestyle. Forest plot showing the ORs and CIs of factors associated with confidence in leading a healthy lifestyle. BTEC, Business and Technology Education Council.

Confidence in the ability to lead a healthy lifestyle was influenced by age, ethnicity, education and health status (figure 2, online supplemental table S4). Older age groups demonstrated greater confidence compared with the 18–24 group, with participants aged 55–64 over two times as likely to report high confidence (aOR=2.11, 95% CI 1.48 to 3.00, p<0.001) and those aged 65+ nearly three times as likely (aOR=2.96, 95% CI 1.87 to 4.68, p<0.001). Ethnic disparities were also observed; Asian British participants were significantly less likely to report confidence than White participants (aOR=0.46, 95% CI 0.34 to 0.63, p<0.001).

Participants with secondary education were less likely to feel confident in leading a healthy lifestyle compared with those with primary education (aOR=0.21, 95% CI 0.05 to 0.89, p=0.034). Similarly, individuals with a disability or a long-term condition reported lower confidence (disability: aOR=0.58, 95% CI 0.45 to 0.74, p<0.001; long-term condition: aOR=0.65, 95% CI 0.55 to 0.77, p<0.001). HCPs were notably more confident in their ability to lead a healthy lifestyle (aOR=1.66, 95% CI 1.24 to 2.23, p=0.001).

### Seeking health information for common conditions

**Figure 3:**
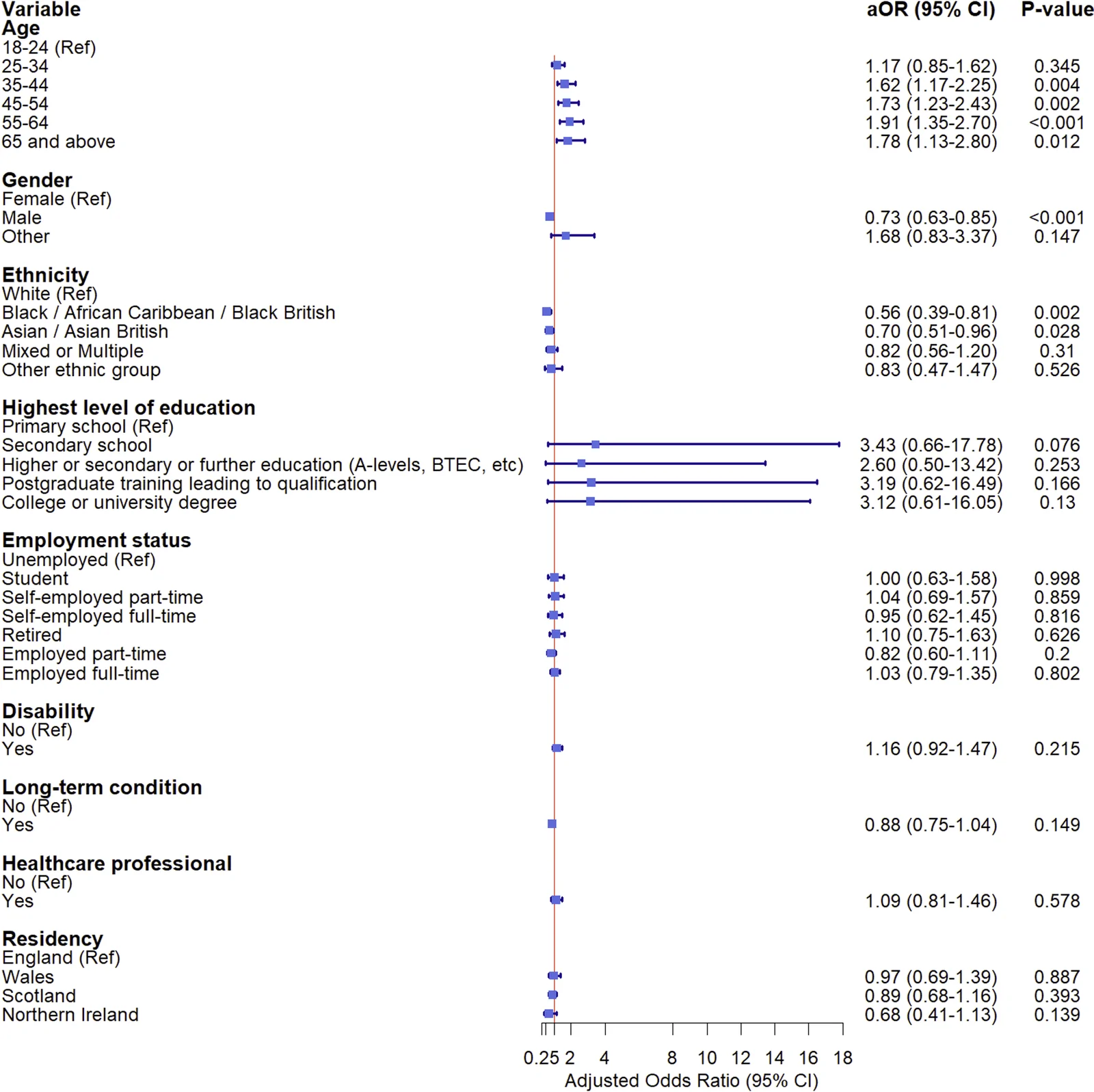
ORs and CIs of factors associated with finding health information when faced with a common condition. Forest plot showing the ORs and CIs of factors associated with finding health information when faced with a common condition. aOR, adjusted odds ratio. BTEC, Business and Technology Education Council.

The ordinal logistic regression analysis (figure 3, online supplemental table S5) explored demographic factors influencing the likelihood of seeking health information when experiencing a common condition. Older participants were more likely to seek health information when experiencing common conditions, with individuals aged 55–64 having a 93% higher likelihood than the 18–24 group (aOR=1.93, 95% CI 1.36 to 2.73, p<0.001). Males were less likely than females to seek health information (aOR=0.73, 95% CI 0.63 to 0.85, p<0.001), while Black and Asian British individuals were less likely than White participants to seek information (Black British aOR=0.56, 95% CI 0.39 to 0.81, p=0.002; Asian British aOR=0.70, 95% CI 0.52 to 0.98, p=0.035).

### Model adequacy and assumption testing

All three models fitted significantly better than the corresponding intercept-only model (self-care knowledge LR χ²=169.6, df=27; healthy lifestyle LR χ²=212.0, df=27; information-seeking LR χ²=97.7, df=27; all p<0.001). McFadden pseudo-R² was 0.028, 0.038 and 0.018 respectively (Cox–Snell 0.055, 0.068 and 0.032; Nagelkerke 0.063, 0.081 and 0.038), with AIC values of 5,962.8, 5423.0 and 5340.7. These values are consistent with models estimating adjusted associations for behavioural outcomes shaped by determinants beyond demographic position. Collinearity between predictor sets was negligible, with all GVIF between 1.01 and 1.13 on the GVIF^∧^(1/2×df) scale. The Brant test indicated departure from the proportional odds assumption in all three models (self-care knowledge χ²=53.31, df=26, p=0.001; healthy lifestyle χ²=39.31, df=26, p=0.046; information-seeking χ²=62.71, df=26, p<0.001). Covariate-specific testing localised these departures. In the self-care knowledge model, five covariates were flagged: Black British ethnicity (χ²=17.67, p<0.001), gender (p=0.016), further education (p=0.016), postgraduate education (p=0.025) and age 45–54 (p=0.030). In the healthy lifestyle model, three were flagged: Northern Ireland residency (p=0.007), disability (p=0.008) and retired status (p=0.049). In the information-seeking model, five were flagged: student status (p=0.002), HCP status (p=0.002), long-term condition status (p=0.007), gender (p=0.022) and age 45–54 (p=0.050). All remaining covariates were consistent with proportional odds.

For flagged covariates that were also significant predictors, threshold-specific estimates preserved the direction and significance of the pooled estimate in all but two instances and preserved statistical significance at both thresholds except in two further instances. Gender and postgraduate education in the self-care knowledge model retained their direction across thresholds but attenuated to non-significance at the highest confidence level, indicating that these differences operate mainly on the likelihood of reporting at least moderate rather than the highest confidence. The two directional exceptions were more consequential. The first was Black British ethnicity in the self-care knowledge model, where the adjusted OR was 0.94 for reporting at least ‘fairly confident’ but 2.57 for reporting ‘very confident’, indicating an association concentrated at the upper end of the confidence distribution rather than uniform across it. The second was Northern Ireland residency in the healthy lifestyle model, where the direction reversed across thresholds; with 69 Northern Irish respondents, this estimate is imprecise and is discussed under limitations.

## Discussion

This study builds on the findings of the LSCS Study,^8^ shifting the focus toward public engagement with self-care behaviours by examining demographic predictors, symptom management strategies and personal barriers to self-care.

### Self-care confidence and health-seeking behaviours

Older adults (65+) were proportionally underrepresented in this online sample, consistent with known digital participation gradients, yet demonstrated the largest and most consistent positive associations with self-care confidence across all domains. This suggests that the true age-related confidence gradient in the broader UK population may be more pronounced than these data indicate. Older adults were also more likely than the youngest group to seek health information when experiencing a common condition, so in this sample confidence and information-seeking moved together rather than diverging. Whether that confidence translates into effective self-care action these data cannot establish and prior research cautions that among older adults, particularly those with multiple long-term conditions, gaps in health literacy or self-efficacy may limit that translation.^25^ Conversely, younger adults (18–34) were overrepresented in the sample yet reported comparatively lower self-care confidence despite being the most digitally engaged group, identifying them as a priority target for foundational self-care literacy interventions. That men reported lower self-care confidence than women is consistent with a substantial literature on men’s reluctance to seek help from health professionals.^26^

In contrast to earlier evidence reporting lower confidence in managing one’s own health among minority ethnic groups in England^27^ and poorer self-management outcomes in South Asian and Black African populations,^28^ our data reveal a more complex picture. The patterns observed across ethnic groups are not uniform and must not be conflated. Black British participants showed a notable paradox: significantly higher self-care confidence alongside a lower likelihood of seeking formal health information, a divergence that may reflect cultural norms, reliance on community or family advice or lower trust in formal healthcare channels—a finding consistent with the wider health literacy literature, which emphasised the interplay between culture, trust and health-seeking behaviour.^29^ Assumption testing sharpens this point: the Black British association with self-care confidence did not satisfy the proportional odds assumption, operating almost entirely on the likelihood of reporting the highest level of confidence rather than uniformly across the scale. The pooled estimate therefore understates how concentrated this difference is at the top of the distribution. By contrast, the ethnicity associations with information-seeking were consistent with proportional odds, so the divergence between confidence and information-seeking in this group holds across the response range. By contrast, Asian British participants showed a congruent pattern of both lower self-care confidence and lower information-seeking, suggesting different underlying barriers potentially related to structural access or linguistic factors. These two groups should not be grouped under the same explanatory framework. The mechanisms underlying these patterns remain speculative in the context of the current main-effects analysis and should be interpreted with caution. Formal testing of interaction terms (eg, ethnicity×age; ethnicity×education) was not undertaken, as the relatively small size of ethnic minority subgroups would generate underpowered and potentially unstable cross-level estimates. Future studies with larger, purposively oversampled ethnic minority cohorts are needed to formally test whether the association between ethnicity and information-seeking varies by age, education or socioeconomic status and to move from descriptive to mechanistic understanding of these patterns.

Additionally, our data suggested that HCPs exhibited significantly greater self-care confidence than the general population, consistent with findings from previous studies on health literacy and professional knowledge translation.^30^ However, the influence of HCPs as self-care advocates appears underutilised, with 20% of participants citing a lack of professional encouragement as a barrier. This supports prior calls for HCPs to take a more active role in promoting self-care.^31^ It should be noted that self-care confidence, as measured in this study, is a single-item self-appraisal that has not been psychometrically validated. The construct may therefore carry qualitatively different meanings across demographic groups, reflecting professionally informed self-efficacy among HCPs, cultural self-reliance among ethnic minority groups, or accumulated life experience among older adults, which may account for some of the paradoxical associations observed. Direct comparisons across these groups should therefore be interpreted with appropriate caution.

### Barriers to self-care and implications for health literacy

The identification of financial constraints (53%) and lack of time (47%) as key barriers highlights the structural challenges that limit self-care engagement, particularly for lower-income and working-age populations. These findings align with existing research highlighting that socioeconomic disparities affect the ability to prioritise preventive health behaviours and access self-care resources. Additionally, a lack of confidence (22%) and limited trust in available health information (19%) suggest that health literacy remains a critical determinant of self-care engagement.

Despite the increasing availability of digital health resources, it was surprising that engagement with online information sources remained low, with under one in 10 symptom episodes prompting participants to go online or check the NHS website. This suggests that digital exclusion, difficulties in navigating online health content and concerns over misconceptions may hinder the effectiveness of digital self-care interventions.^32^ Collectively, these insights highlight the urgency of embedding health equity principles into national self-care strategies, particularly given the current emphasis on health system sustainability and population resilience.

### Symptom management strategies and health and care resource utilisation

The study highlights a strong inclination toward self-care, with OTC medication use and passive waiting being the dominant management strategies for most symptoms. While this suggests confidence in managing minor ailments, the low rates of engagement with pharmacists and HCPs indicate potential gaps in accessing expert guidance when needed. Given that<2% of participants consulted a pharmacist for symptom management, there is a missed opportunity to leverage pharmacists as accessible self-care advisors as recommended by national self-care policy frameworks.

Additionally, around 2% of respondents sought GP consultations for most symptoms, except for COVID-19 symptoms (8%) and back pain (3%), suggesting that individuals predominantly reserve health and care visits for perceived severe or persistent conditions. While avoiding unnecessary GP visits aligns with self-care promotion goals, there is a need to ensure that individuals can recognise when professional intervention is necessary. Targeted health literacy initiatives could help individuals differentiate between self-treatable conditions and symptoms requiring medical attention, reducing both avoidable consultations and health risks associated with delayed care.

### Symptom iceberg

The findings of this study reinforce and extend existing evidence regarding self-care behaviours, symptom management strategies and barriers to engagement in the UK population. Notably, our results support the foundational work by Elliott *et al*^22^ who in 2011 described the persistence of the ‘symptom iceberg’ phenomenon, where the majority of common symptoms are managed privately rather than presented to primary care.^22^ Our study, conducted over a decade later, suggests a marked intensification of this pattern, with over 90% of respondents managing symptoms independently, largely due to the use of OTC medicines increasing from 25.0% to 51.3% and minimal engagement with pharmacists (1.3%) or GPs (2.1%) across most conditions. Compared with Elliott’s estimate of 45% self-care for symptoms such as headaches or sore throats, our findings indicate a significant upward shift, although the underlying drivers of empowerment, digital information access and constrained service availability require further exploration. Comparatively, our findings also suggest a decrease in the ‘tip of the iceberg’, with consultations with any professional reducing from 13.2% to 5.3%. Consultations with GPs reduced from 8.3% to 2.1% and with pharmacists from 1.8% to 1.3%.

### Health services utilisation

These findings also align with contemporary analyses of changing health service utilisation. Ladds *et al*^33^ note the shift toward remote assessment and digital-first models, particularly following COVID-19, which may partly explain reduced engagement with face-to-face professional advice. However, our findings challenge the assumption that digital uptake is universal; engagement with digital resources (eg, NHS websites, symptom checkers) remained below 10%, especially among older and lower socioeconomic groups.^34 35^ This reflects earlier health literacy research which identified persistent gaps in the functional and interactive skills required to engage with digital health tools effectively.^30 36^

### Study implications

This study provides fresh and critical insights into how UK adults manage common health symptoms, highlighting persistent demographic disparities and deeply rooted structural barriers to self-care. The data highlights the continued relevance and evolution of the ‘symptom iceberg’ in today’s digitally connected yet unevenly accessible health landscape. These findings have significant implications for health system strategy, public health policy and service design, particularly in the context of preventive care, digital transformation and health equity.

The widespread engagement with self-care behaviours revealed in this study—especially reliance on OTC medications and passive symptom monitoring—suggests that the UK public is already navigating a large proportion of their health concerns outside the formal care system. However, structural and demographic barriers such as financial constraints, lack of time, low confidence and digital exclusion disproportionately limit self-care access and efficacy for key population groups, particularly people living with long-term conditions, disabilities and those from ethnic minority backgrounds. These disparities position self-care not simply as a matter of individual choice but as a structural and social justice issue requiring systemic intervention.

Despite strong policy endorsement of self-care, this study shows that few individuals turn to professionals—particularly pharmacists—for support. Engagement with pharmacists for symptom management remained at or below 2% across nearly all symptoms. This underutilisation highlights a missed opportunity. Community pharmacies are well-placed to serve as accessible hubs for self-care guidance, but this will require strategic investment in pharmacist training, remuneration for self-care consultations and public education to shift perceptions of pharmacists as self-care enablers.

An important paradox emerging from this study is the mismatch between reported self-care confidence and information-seeking behaviour among Black British respondents, who reported the highest confidence in their self-care knowledge yet were among the least likely to seek health information when unwell. These findings highlight the limitations of assuming confidence alone predicts behaviour. Health literacy interventions must go beyond general messaging and target the underlying drivers of this disconnect: motivational beliefs, information appraisal skills, cultural perceptions of care and trust in available sources. Initiatives should include co-designed content, culturally competent materials and trusted messengers embedded in local communities.

While digital health is increasingly central to self-care promotion, our data show that the NHS website was consulted in only 3.1% of symptom episodes and any online source in 9.7% This low engagement challenges the assumption that digital-first strategies are reaching or resonating with the public. Digital health content must be reimagined for accessibility linguistically, culturally and functionally. This includes plain language, intuitive design, mobile-first interfaces and embedded signposting to real-world support (eg, community pharmacies, local self-care workshops). National strategies should also fund digital navigation roles and peer educators to improve confidence in using these tools.

The expanded symptom iceberg we observed—with over 90% of common conditions managed independently—raises concern about missed or delayed care, particularly among populations with low self-efficacy or limited access to guidance. Policymakers and public health agencies should promote structured symptom triage tools (digital or paper-based) that guide users through symptom severity, duration and red flags, linking them to the appropriate level of care. These tools should be embedded into public health campaigns and digital portals and integrated within pharmacy and GP reception settings.

Given the variability in self-care confidence and engagement, there is a strong case for incorporating brief self-care readiness assessments into existing patient pathways—such as NHS Health Checks, chronic disease reviews or vaccination appointments. These tools can help clinicians identify individuals who may benefit from targeted support, including health coaching, literacy interventions or peer navigation. A standardised tool, derived from items used in this study, could enable scalable, comparable assessments of self-care capability across settings and populations.

The dominant barriers to self-care identified—money, time, confidence, information and access—span beyond the health sector and demand joined-up action. Government and local authorities should work in partnership with employers, housing providers, education institutions and third-sector organisations to build supportive ecosystems for self-care. Initiatives could include flexible workplace well-being schemes, self-care skills in school curricula, social prescribing for digital literacy training and voucher schemes to subsidise self-care essentials like thermometers or OTC medications for low-income households.

With mounting pressures on general practice and urgent care, the findings support the development of a national symptom management strategy that explicitly outlines how common, self-limiting conditions can be safely managed by the public with professional support as needed. This would include clear care pathways, a public-facing symptom reference guide and greater investment in communications that empower people to care for themselves while knowing when—and how—to seek help.

### Limitations

This study has several limitations that were acknowledged in the first paper.^8^ Briefly, the reliance on self-reported data introduces the possibility of recall bias and social desirability effects, particularly in the reporting of health behaviours, confidence and symptom management strategies. Although the survey was anonymous, participants may have over- or under-estimated their engagement with self-care or health information-seeking behaviours. Second, the cross-sectional design limits causal inference. While we identified significant associations between demographic variables and self-care engagement, we cannot determine whether these factors directly influence self-care behaviour or whether unmeasured variables, such as prior health experiences, social support or mental health status, may mediate these relationships. Third, the study sample was recruited by open, non-probability methods and is not representative of the UK adult population. Adults aged 65 and over were substantially under-represented (9.4% of the sample), and respondents educated to degree level or above were over-represented (65.8%). In comparison, the gender distribution (52.1% female) and broad ethnic group distribution (82.4% White) were closer to national figures. We therefore report associations within the sample rather than population estimates. Individuals with limited digital access, lower levels of digital literacy or those disengaged from online health discourse may have been under-represented. As a result, estimates of engagement with digital health resources and confidence in self-care may be biased toward more digitally literate respondents. Also, the absence of an a priori power calculation means that analyses for smaller demographic subgroups may be underpowered; replication in larger, purposively sampled studies is recommended. Fourth, the proportional odds assumption was not met in any of the three models. Covariate-specific testing showed the departures to be confined to a minority of terms, most of which were not significant predictors or retained both direction and significance across thresholds. Gender and postgraduate education in the self-care knowledge model retained their direction but were significant only at the lower threshold. Two further exceptions warrant note: Black British ethnicity in the self-care knowledge model, whose association is concentrated at the highest confidence category, and Northern Ireland residency in the healthy lifestyle model, where the direction reversed across thresholds on only 69 respondents. Relatedly, only seven respondents reported primary school as their highest attainment, so the education comparisons rest on a very small reference category and yield unstable estimates with wide CIs; these should not be over-interpreted. Fifth, the variation explained by demographic characteristics alone was modest. These models were specified to estimate adjusted associations rather than to predict individual behaviour, so the ORs describe average differences between demographic groups; self-care engagement is shaped by factors we did not capture, including health beliefs, social support, prior healthcare experience and local service availability, and these should be examined in future work. Sixth, while our regression models adjusted for a wide range of demographic variables, residual confounding cannot be excluded. For instance, variables such as income, health insurance status (eg, private vs NHS-only care), caregiving responsibilities or migration status were not included but may influence self-care capability and behaviour. Additionally, HCP status was included as a covariate in pooled models rather than as a basis for stratification; self-care confidence likely carries a different construct meaning for professionals than for the general public, and this may have affected model interpretation. Finally, although the study explored a range of barriers to self-care, it did not include qualitative data to contextualise individual choices or explore the influence of cultural beliefs, trust in health systems or lived experiences of marginalisation. Future mixed-methods research is needed to deepen our understanding of the drivers behind observed disparities in self-care engagement.

## Conclusion

This study highlights demographic disparities in self-care engagement, emphasising the influence of confidence, health literacy, financial constraints and access to reliable health information on self-care behaviours. While self-care was the preferred approach for managing common symptoms, low engagement with pharmacists, digital resources and professional health and care support suggests that further efforts are needed to optimise self-care literacy and accessibility. Addressing structural barriers, enhancing pharmacist roles and improving digital health strategies are essential to ensuring that self-care initiatives effectively reach all population groups. Future research should explore interventions that enhance self-care confidence, reduce inequalities and support sustainable health and care models.

## Supplementary material

10.1136/bmjopen-2025-110380 online supplemental file 1

10.1136/bmjopen-2025-110380 online supplemental file 2

10.1136/bmjopen-2025-110380 online supplemental file 3

10.1136/bmjopen-2025-110380 online supplemental file 4

10.1136/bmjopen-2025-110380 online supplemental file 5

10.1136/bmjopen-2025-110380 online supplemental file 6

## Data Availability

All data relevant to the study are included in the article or uploaded as supplementary information.
