# Supplementary material for: What are the demographic predictors and barriers to self-care engagement in the UK? a cross-sectional survey study
Source: BMJ Open. 2026 Sep 21;16(9):e110380. doi: 10.1136/bmjopen-2025-110380 (PMC13599892; doi:10.1136/bmjopen-2025-110380)
Supplement: Supplementary file 1 [file bmjopen-2025-110380.reviewer_comments.pdf]

PEER REVIEW HISTORY

BMJ Open publishes all reviews undertaken for accepted manuscripts. Reviewers are asked to complete a checklist review form and are provided with free text boxes to elaborate on their assessment. These free text comments are reproduced below.

ARTICLE DETAILS

Title (Provisional)

What are the Demographic Predictors and Barriers to Self-Care Engagement in the UK? A Cross-Sectional Survey Study

Authors

El-Osta, Austen; Altalib, Sami; Al Ammouri, Mahmoud; Smith, Peter

---

VERSION 1 - REVIEW

**Reviewer**	**1**
**Name**	**Peimani , Maryam**
**Affiliation**	**Tehran University of Medical Sciences, Diabetes Research Center, Endocrinology and Metabolism Clinical Sciences Institute**
**Date**	**06-Jan-2026**
**COI**	**none**

The study presents a cross-sectional analysis of an online survey of 3,255 UK adults, aiming to identify demographic predictors (age, gender, ethnicity, professional status, etc.) of self-care engagement and perceived barriers. Key findings include higher self-care confidence among older adults and Black British participants, lower confidence among males and those with disabilities or long-term conditions, and low utilization of pharmacists and digital NHS resources despite high reported confidence. Financial constraints, lack of time and low self-efficacy emerged as dominant barriers.

The novelty of this work lies in its large-scale examination of self-care disparities in a UK context, incorporating health professionals as a subgroup and building on the authors' prior Living Self-Care Survey (Smith et al., 2025) to provide granular insights into symptom management for common ailments and barriers, adding value beyond existing literature that often focuses on narrower populations or lacks integration of professional perspectives, thereby informing targeted interventions for health equity. However, this novelty is significantly overstated and inadequately justified. The introduction and methods repeatedly reference Smith et al., 2025 for full methodological details, making this manuscript read as a secondary analysis or a companion paper rather than a standalone novel contribution. The authors must explicitly and quantitatively define what new research questions are addressed

here that were not in the prior publication. The abstract and introduction fail to establish a clear incremental knowledge gap that this analysis fills, beyond a general shift in "focus." A more precise articulation of novelty—for instance, specific hypothesis testing about demographic interactions, deeper analysis of barriers by subgroup, or novel application of statistical models to this dataset—is required.

The sample size of 3,255 is commendably large and enhances statistical power, yet there is no justification provided for this number; no a priori power calculation is mentioned to determine if it was sufficient to detect meaningful effects across subgroups, such as ethnic minorities or those with disabilities, which could lead to underpowered analyses for rarer demographics. Recruitment via online platforms and professional networks introduces substantial selection bias, as noted in the limitations, potentially overrepresenting digitally literate, health-engaged individuals and underrepresenting vulnerable groups like those with low socioeconomic status or limited internet access—this seriously undermines the claim of a "nationally representative sample" in the strengths section.

The inclusion of a subset of "health and care professionals" (HCPs) is a potential confounder rather than a simple predictor. The analysis treats HCP status as a demographic variable akin to ethnicity, but this is problematic. HCPs possess expert knowledge that fundamentally changes the meaning of "self-care confidence" and health information-seeking. Comparing them directly to the lay public without acknowledging this fundamental difference in construct validity weakens the interpretation. The analysis should either: a) stratify all models by HCP status, presenting separate results for public and professional populations, or b) exclude HCPs from the primary analysis of public behavior and analyze them as a separate comparator cohort. Their current treatment is overly simplistic.

The reliance on self-reported, non-validated measures for key outcomes like "confidence" is a limitation appropriately noted but necessitates greater caution in interpretation. The construct of "self-care confidence" is loosely defined and may encompass different meanings across demographic groups, potentially explaining some paradoxical findings.

A significant issue in statistical analysis is the lack of investigation into effect modification (interactions). The most intriguing and potentially novel findings are the paradoxical results: e.g., Black British individuals report higher confidence but are less likely to seek formal health information. Similarly, older adults are more confident but also more likely to seek information. These contradictions beg for statistical testing of interactions (e.g., does the association between ethnicity and information-seeking vary by age or education?). The current analysis only presents main effects, leaving these critical questions unexplored and the discussion speculative. Adding interaction terms to the models or presenting stratified analyses is necessary to move beyond description to mechanistic insight.

Specific comments

-Page 5, Lines 25-30; Page 6, Lines 1-5: The "critical gap" identified is reasonable. However, the link to the authors' prior work (Smith et al., 2025) creates ambiguity. What specific,

unanswered questions from that prior study does this manuscript address? This must be explicitly stated. Currently, the aim (to build on initial findings... by exploring demographic predictors...) is vague and does not convincingly establish standalone novelty.

-Page 6, Methods, Study design: “This study is part of the Living Self-Care Survey (LSCS) Study..." – Cross-reference to Smith et al., 2025 is fine, but please provide key details here for standalone readability, e.g., response rate.

-Page 6, Measures and variables: “This study examines self-care engagement through five key domains..." – Domains are listed, but operational definitions (e.g., how "self-care confidence" was measured) are absent; include in supplementary or text.

-Page 6, Statistical analysis: The statistical analysis description is adequate but must include mention of how missing data were handled (e.g., complete-case analysis) and the justification for the chosen reference categories (e.g., why "White" as reference for ethnicity? A more equitable approach might be using the largest group, but justification is needed).

-The contrasting patterns for ethnic groups are a key finding. For Black British participants, there is a paradoxical pattern of higher reported self-care knowledge confidence yet a lower likelihood of seeking health information. For Asian British participants, the pattern is congruent: lower confidence and lower information-seeking. The authors' discussion (Page 12) erroneously groups both groups under the "higher confidence" paradox, which is not supported by their data for Asian British respondents. This conflation must be corrected. Furthermore, these findings, particularly the Black British paradox, demand deeper analysis. The authors should test for interactions (e.g., ethnicity x age, ethnicity x education) to explore whether the association between confidence and information-seeking differs across demographic strata, moving beyond main effects.

**Reviewer**	**2**
**Name**	**Mehta, KM**
**Affiliation**	**University of California San Francisco**
**Date**	**09-Feb-2026**
**COI**	**none**

This is a cross-sectional study of self-reported self care engagement in the United Kingdom. The respondents span a range of age and demographic characteristics, though the survey was taken only online which may limit generalizability. The process of self care engagement is important for health care and the need for self care engagement continues to increase as the health care system is increasingly expensive, hard to access and health care worker shortages abound. Thus this article is written on an important topic.

Overall, the background does not address the novely of the research questions posed nor does it state the research questions plainly. Please consider a more thorough literature review and compare and contrast the measures used in this survey to other surveys and constructs that are closely related.

Several places in the manuscript could be strengthend. Overall, the methods are not written in sufficient detail to replicate the findings. I would encourage the authors to add a great deal of detail, including the specification for all models.

- Overall the age range is young, but the effects are seen in the older participants. What do the authors make of this finding?

- Clarify the definition of self care confidence. How is it different from other constructs in health such as self efficacy

- Do you have data or substantiation for whether a physician corroborates the diagnoses or are these all self-reported diagnoses?

- Do you have data on over the counter products used? If so, are these evidence based therapies for the conditions? Is there any way to measure this? If not, please state as a limitation.

- Does the amount of self care confidence vary by condition? This would actually be very interesting to note? Did you consider a stratified analyses?

- Given that financial limitations seem to track with self care confidence, do the authors think that it is actually an effect of confidence or of finances? What is the relationship between these two, interrelated constructs? Please discuss.

-Table 1 is very difficult to read and poorly formatted

-Figure 1 does nto make sense as presented. Is it actually true that higher education is associated with lower self care confidence? This may be miscoded, please double check.

- On finding healht ifnormation, all of these confidence intervals seem to cross 1. There are no true effects present.

- Do you have any information on the combination of health information sought? That coudl be potentially very interesting.

It is unclear what the referent category is in most models and categorical analyses presented. Please revise and add many more details on the exact questions asked and how the variables were parameterized and used in the analyses. Please itnerpret the odds using the 95% confidence intervals not the point estimate.

One of the main weaknesses of the paper is that none of these conditions have been confirmed by a MD. Another main weakness is the lack of details of the methods used. If these two can either be addressed or discussed, I think this could make a nice contribution

to the field. Another key would be to use any of the analyses as insight on how public health practitioners can increase self-care engagement and confidence in the future.

VERSION 1 - AUTHOR RESPONSE

<table>
<tr><th>Reviewer 1</th><th></th></tr>
<tr><td>The study presents a cross-sectional analysis of an online survey of 3,255 UK adults, aiming to identify demographic predictors (age, gender, ethnicity, professional status, etc.) of self-care engagement and perceived barriers. Key findings include higher self-care confidence among older adults and Black British participants, lower confidence among males and those with disabilities or long-term conditions, and low utilization of pharmacists and digital NHS resources despite high reported confidence. Financial constraints, lack of time and low self-efficacy emerged as dominant barriers.</td><td>We thank you for the accurate and balanced summary of the study and for recognising the importance of this topic.</td></tr>
<tr><td>The novelty of this work lies in its large-scale examination of self-care disparities in a UK context, incorporating health professionals as a subgroup and building on the authors' prior Living Self-Care Survey (Smith et al., 2025) to provide granular insights into symptom management for common ailments and barriers, adding value beyond existing literature that often focuses on narrower populations or lacks integration of professional perspectives, thereby informing targeted interventions for health equity. However, this novelty is significantly overstated and inadequately justified.

The introduction and methods repeatedly reference Smith et al., 2025 for full methodological details, making this manuscript read as a secondary analysis or a companion paper rather than a standalone novel contribution. The authors must explicitly and quantitatively define what new research questions are addressed here that were not in the prior publication.

The abstract and introduction fail to establish a clear incremental knowledge gap that this analysis fills, beyond a general shift in "focus." A more precise articulation of novelty—for instance, specific hypothesis testing about demographic interactions, deeper analysis of barriers by subgroup, or novel application of statistical models to this dataset—is required.</td><td>We thank the reviewer for this important point. We agree that the framing required strengthening. Paper 1 (Smith et al., 2025) examined the interplay between self-care confidence, professional support and health literacy, specifically the public–HCP interface, barriers perceived by professionals, and health literacy measured using the validated HLS19-Q12 instrument.

This second paper addresses three specific questions that were not examined in Paper 1:
(i) which demographic characteristics independently predict self-care confidence and health information-seeking behaviour in the general UK public;
(ii) What are the most prevalent personal and structural barriers to self-care engagement as reported by the public?
(iii) how do symptom management strategies and professional resource utilisation vary by symptom type across the population?

We have revised the Introduction to make this distinction explicit and have rewritten the aim statement accordingly.</td></tr>
<tr><td>The sample size of 3,255 is commendably large and enhances statistical power, yet there is no justification provided for this</td><td>We acknowledge this limitation. The sample size of 3,255 was determined by the feasibility of online recruitment over the study period and not by</td></tr>
</table>

number; no a priori power calculation is mentioned to determine if it was sufficient to detect meaningful effects across subgroups, such as ethnic minorities or those with disabilities, which could lead to underpowered analyses for rarer demographics.	formal a priori power calculation, consistent with the approach taken in Paper 1, which also describes this as a pragmatic convenience sample. We have added this clarification to the Methods and noted in the Limitations that subgroup analyses for smaller ethnic groups should be interpreted with caution.
Recruitment via online platforms and professional networks introduces substantial selection bias, as noted in the limitations, potentially overrepresenting digitally literate, health-engaged individuals and underrepresenting vulnerable groups like those with low socioeconomic status or limited internet access—this seriously undermines the claim of a "nationally representative sample" in the strengths section.	Thank you for this important point. We agree that recruitment via online platforms and professional networks introduces selection bias and limits the extent to which the sample can be considered nationally representative. We have revised the manuscript to remove the claim of a “nationally representative sample” from the strengths section and to more clearly characterise the sample as a large, diverse, non-probability sample. We also expanded the limitations to explicitly acknowledge the potential overrepresentation of digitally literate and health-engaged individuals and the underrepresentation of more vulnerable groups, including those with lower socioeconomic status or limited internet access.
The inclusion of a subset of "health and care professionals" (HCPs) is a potential confounder rather than a simple predictor. The analysis treats HCP status as a demographic variable akin to ethnicity, but this is problematic. HCPs possess expert knowledge that fundamentally changes the meaning of "self-care confidence" and health information-seeking. Comparing them directly to the lay public without acknowledging this fundamental difference in construct validity weakens the interpretation. The analysis should either: a) stratify all models by HCP status, presenting separate results for public and professional populations, or b) exclude HCPs from the primary analysis of public behavior and analyze them as a separate comparator cohort. Their current treatment is overly simplistic.	We agree that this is an important conceptual observation. In Paper 1, HCP-specific items were administered in a dedicated, separate survey section (n=227), and HCP status was included as a covariate in pooled models. Paper 2 adopts the same analytical framework for consistency. We have revised the relevant text to clarify that HCP status functions as a comparator variable rather than a primary demographic predictor, and we have added appropriate caveats in the Discussion and Limitations.
The reliance on self-reported, non-validated measures for key outcomes like "confidence" is a limitation appropriately noted but necessitates greater caution in interpretation. The construct of "self-care confidence" is loosely defined and may encompass different meanings across demographic groups, potentially explaining some paradoxical findings.	We thank you for this important point. We agree that greater caution is warranted in interpreting the self-care confidence findings, and that the construct may carry different meanings across demographic groups, potentially explaining some of the paradoxical patterns observed. We have added language throughout the Results and Discussion to temper inferential claims, and have acknowledged the single-item, non-validated nature of the confidence measure more prominently in both the Methods and Limitations sections.
A significant issue in statistical analysis is the lack of investigation into effect modification (interactions). The most intriguing and potentially novel findings are the paradoxical results: e.g., Black British individuals report	We thank you for this suggestion. However, we submit that adding interaction terms to these models would not meaningfully advance the analytical objectives of this paper for the following reasons.

higher confidence but are less likely to seek formal health information. Similarly, older adults are more confident but also more likely to seek information. These contradictions beg for statistical testing of interactions (e.g., does the association between ethnicity and information-seeking vary by age or education?). The current analysis only presents main effects, leaving these critical questions unexplored and the discussion speculative. Adding interaction terms to the models or presenting stratified analyses is necessary to move beyond description to mechanistic insight.	First, the primary aim of this study is descriptive and epidemiological, to identify the independent demographic predictors of self-care engagement at a population level, rather than to establish causal or mechanistic pathways. Main-effects regression is the appropriate and sufficient analytical tool for this objective.<br><br>Second, the statistical conditions for interaction testing in this dataset are constrained. Several ethnic minority subgroups, particularly Black British (n=154, 4.7%) and Asian British (n=213, 6.5%), are relatively small. Introducing ethnicity × age or ethnicity × education interaction terms would generate a large number of cross-level cells with very small counts, substantially reducing statistical power, inflating standard errors, and producing unstable estimates that would be difficult to interpret meaningfully. The risk of spurious interaction effects under these conditions is considerable.<br><br>Third, the "paradoxical" patterns observed, such as Black British participants reporting higher confidence but lower information-seeking, are already discussed in the context of established theoretical frameworks (cultural norms, trust in formal health systems, community health-seeking behaviour). This interpretive framing does not require interaction testing to be valid; it is consistent with existing qualitative and sociological evidence in the literature.<br><br>Nevertheless, we recognise the reviewer's underlying concern that the discussion of these paradoxical findings should not overreach what the main-effects models can support. We have therefore revised the Discussion to more explicitly qualify interpretations of the Black British and older adult paradoxes as hypothesis-generating rather than confirmatory, and have added a clear recommendation for future studies, ideally with larger, purposively oversampled ethnic minority cohorts, to formally test these interactions with adequate statistical power.
-Page 5, Lines 25-30; Page 6, Lines 1-5: The "critical gap" identified is reasonable. However, the link to the authors' prior work (Smith et al., 2025) creates ambiguity. What specific, unanswered questions from that prior study does this manuscript address? This must be explicitly stated. Currently, the aim (to build on initial findings... by exploring demographic predictors...) is vague and does not convincingly establish standalone novelty.	Please refer to our response to the first comment above, where the aim statement has been rewritten to enumerate three explicit research questions.
-Page 6, Methods, Study design: "This study is part of the Living Self-Care Survey (LSCS) Study..." – Cross-reference to Smith et al., 2025 is fine, but please provide key details	Please see our response to the Editor's third comment above, where the Methods section has been expanded with all required details drawn directly from Paper 1 for the paper to read as a

here for standalone readability, e.g., response rate.	standalone. Also, the absence of a response rate was justified.
-Page 6, Measures and variables: “This study examines self-care engagement through five key domains..." – Domains are listed, but operational definitions (e.g., how "self-care confidence" was measured) are absent; include in supplementary or text.	Thank you for your comment. In addition to what was edited in the previous comment, wider operational definitions have been added to the Measures and Variables subsection.
-Page 6, Statistical analysis: The statistical analysis description is adequate but must include mention of how missing data were handled (e.g., complete-case analysis) and the justification for the chosen reference categories (e.g., why "White" as reference for ethnicity? A more equitable approach might be using the largest group, but justification is needed).	We thank the reviewer for this comment. We have now added explicit details on missing data handling and provided full justification for all reference categories chosen across the regression models. Reference categories were selected based on either the largest group in the sample or the most commonly used comparator in the existing UK health disparities literature.
-The contrasting patterns for ethnic groups are a key finding. For Black British participants, there is a paradoxical pattern of higher reported self-care knowledge confidence yet a lower likelihood of seeking health information. For Asian British participants, the pattern is congruent: lower confidence and lower information-seeking. The authors' discussion (Page 12) erroneously groups both groups under the "higher confidence" paradox, which is not supported by their data for Asian British respondents. This conflation must be corrected.<br><br>Furthermore, these findings, particularly the Black British paradox, demand deeper analysis. The authors should test for interactions (e.g., ethnicity x age, ethnicity x education) to explore whether the association between confidence and information-seeking differs across demographic strata, moving beyond main effects.	We sincerely thank the reviewer for identifying this. Our data show that Asian British participants reported lower self-care confidence (aOR=0.66, 95% CI: 0.49–0.89, p=0.006), a pattern congruent with their lower information-seeking (aOR=0.70), not paradoxical. The paradox applies exclusively to Black British participants (higher confidence aOR=1.99; lower information-seeking aOR=0.56).<br><br>We have corrected this. Regarding the request for interaction testing, please see our response and justification above.
**Reviewer 2**	
This is a cross-sectional study of self-reported self care engagement in the United Kingdom. The respondents span a range of age and demographic characteristics, though the survey was taken only online which may limit generalizability. The process of self care engagement is important for health care and the need for self care engagement continues to increase as the health care system is increasingly expensive, hard to access and health care worker shortages abound. Thus this article is written on an important topic.	We thank you for acknowledging the importance of this topic and for the constructive and detailed feedback.
Overall, the background does not address the novely of the research questions posed nor does it state the research questions plainly. Please consider a more thorough literature review and compare and contrast the	We thank you for this comment. We would like to clarify an important contextual point: this is the second in a paired series of papers from the Living Self-Care Survey (LSCS) Study. The first paper established the survey instrument through a

measures used in this survey to other surveys and constructs that are closely related.	rigorous development process involving literature review, adaptation of validated tools, including the HLS19-Q12, the 2005 Department of Health Self-Care Baseline Survey and Elliott et al.'s Symptom Iceberg studies, expert consultation and piloting. It also provided a detailed comparison of the survey's constructs to related measures in the literature, including health literacy, self-management and professional guidance frameworks. To avoid redundancy, the Introduction of this paper was intentionally concise in its literature review, with the expectation that both papers would be read as a series.<br><br>We nonetheless accept that this paper must stand alone and that the novelty of its specific research questions must be explicitly stated. We have therefore revised the Introduction to: (i) briefly situate this paper's contribution relative to the first paper; (ii) state three explicit research questions addressed here that were not examined in the first paper; and (iii) add a paragraph distinguishing self-care confidence from related constructs, condensed from the conceptual framework established in the first paper.
Several places in the manuscript could be strengthend. Overall, the methods are not written in sufficient detail to replicate the findings. I would encourage the authors to add a great deal of detail, including the specification for all models.	We thank the reviewer and fully accept this criticism. The Methods section was written concisely because this paper is part of the Living Self-Care Survey (LSCS) Study series, but we recognise it must be self-contained. We have substantially expanded the Methods section with all the details required.
- Overall the age range is young, but the effects are seen in the older participants. What do the authors make of this finding?	We thank the reviewer for this observation. The online survey design naturally overrepresents younger, digitally engaged adults, a known limitation of web-based convenience sampling. Despite this, older adults demonstrated the strongest and most consistent positive associations with self-care confidence across all outcome domains. This finding has two important implications. First, our estimates for older adults may in fact be conservative; if a more representative sample were achieved, the age-related confidence gradient could be even more pronounced. Second, and perhaps more actionably, younger adults (18–34), overrepresented in this sample, reported comparatively lower self-care confidence despite being the most digitally engaged group, identifying them as a priority target for foundational self-care literacy interventions. We have added a paragraph to the Discussion addressing this.
- Clarify the definition of self care confidence. How is it different from other constructs in health such as self efficacy	Self-care confidence in this study is a global, non-condition-specific self-appraisal of one's ability to manage common health conditions independently. This differs from self-efficacy, which is task-specific and domain-specific, typically measured using validated multi-item instruments. It also differs from patient activation, which focuses on

	engagement with formal healthcare, and from health literacy, which concerns the ability to access and appraise health information. Our measure is broader in scope but gains breadth at the cost of psychometric precision, which we now acknowledge explicitly.
- Do you have data or substantiation for whether a physician corroborates the diagnoses or are these all self-reported diagnoses?	In the section on behaviours, as in other research papers on this topic (note McAteer) symptoms are used rather than conditions to avoid the need for professional confirmation. Where health conditions, disabilities and symptoms are referred to they are entirely self-reported. No physician confirmation was conducted. We note, however, that self-reported health status is the accepted standard methodology in large-scale population surveys, including the Health Survey for England and the GP Patient Survey. Physician verification at this scale is neither logistically nor ethically feasible. We have clarified this in the methods that all reported health conditions, disabilities and symptoms are self-reported and have not been clinically verified.
- Do you have data on over the counter products used? If so, are these evidence based therapies for the conditions? Is there any way to measure this? If not, please state as a limitation.	The use of OTC medications is itself a recognised and legitimate component of self-care behaviour, and capturing its prevalence was a primary objective of this study. The survey recorded OTC medication use as a binary management strategy per symptom, confirming that respondents engaged in this form of self-care. However, the investigation of whether the specific products selected were evidence-appropriate for the reported conditions is a distinct and more granular research question that falls beyond the scope of this study. Furthermore, capturing product-level data at scale in web surveys is methodologically challenging due to brand recognition variability and generic equivalence.
- Does the amount of self-care confidence vary by condition? This would actually be very interesting to note? Did you consider a stratified analyses?	Self-care confidence was operationalised as a global, non-condition-specific construct, which precludes formal stratification by diagnosis. However, the symptom management data in Table 1 provide indirect evidence of condition-specific behavioural variation, for example, high passive waiting for back pain (42.7%) and joint pain (53.0%) contrasted with high OTC use for headaches (71.9%), suggesting that condition-level confidence differences likely exist.
- Given that financial limitations seem to track with self-care confidence, do the authors think that it is actually an effect of confidence or of finances? What is the relationship between these two, interrelated constructs? Please discuss.	These constructs are likely mutually reinforcing. Financial constraints limit access to OTC products, healthy food and exercise, eroding the material basis for self-care and potentially undermining confidence. Conversely, low confidence may suppress self-care attempts, reducing the experiential learning that builds self-efficacy. The cross-sectional design precludes conclusions about directionality. We have added a paragraph to the Discussion addressing this bidirectionality.
-Table 1 is very difficult to read and poorly formatted	We thank the reviewer for this feedback. We have removed Table 1 entirely. The same data, fully

	verified and correctly formatted, are already presented in Supplementary Table 2, which we consider sufficient for the reader's reference alongside the narrative description in the Results section.
-Figure 1 does not make sense as presented. Is it actually true that higher education is associated with lower self care confidence? This may be miscoded, please double check.	We thank the reviewer for flagging this. The apparent inverse education gradient is a statistical artefact: the reference category (primary school, n=7, 0.2%) is extremely small, yielding highly unstable estimates with wide confidence intervals across all education comparisons. Variable coding has been verified and no miscoding was identified. We have added an explicit caveat to the Results.
- On finding healht ifnormation, all of these confidence intervals seem to cross 1. There are no true effects present.	This is correct. Where 95% CIs include 1.0, associations are not statistically significant. Also, where the 95% CIs cross the 1.0, the p-value will be >0.05..<br>Several variables in the adjusted model show statistically significant associations with 95% CIs that do not cross 1.0: older age groups (35+), male gender, Black British ethnicity and Asian British ethnicity all show significant adjusted associations ($p<0.05$). However, that education, employment, disability, long-term condition, HCP status and residency all show adjusted CIs crossing 1.0, confirming no statistically significant association for those predictors.
- Do you have any information on the combination of health information sought? That coudl be potentially very interesting.	We agree that this is a valuable direction. The survey used multiple-response items, so co-selection patterns can be examined. A formal and deeper analysis of information-seeking profiles is beyond the scope of this paper.
It is unclear what the referent category is in most models and categorical analyses presented. Please revise and add many more details on the exact questions asked and how the variables were parameterized and used in the analyses. Please itnerpret the odds using the 95% confidence intervals not the point estimate.	We thank the reviewer for this feedback.<br>Reference categories were stated in the Statistical Analysis section of the Methods and in the tables (supplementary files).<br>95% confidence intervals are already reported numerically in parentheses alongside every point estimate throughout the Results section, supplementary tables and figures, and are therefore available to the reader for every association. We have ensured that the narrative explicitly flags where CIs cross 1.0 and associations should not be interpreted as statistically significant, which we believe represents a practical and readable approach to CI-informed interpretation.
One of the main weaknesses of the paper is that none of these conditions have been confirmed by a MD. Another main weakness is the lack of details of the methods used. If these two can either be addressed or discussed, I think this could make a nice contribution to the field. Another key would be to use any of the analyses as insight on how public health practitioners can increase self-care engagement and confidence in the future.	Both weaknesses are addressed above: clinical verification is stated in Methods; Methods have been fully expanded.<br><br>On public health implications: The Study implications section has been substantially developed to translate findings directly into practitioner-facing recommendations, including: investment in accessible symptom triage tools; repositioning community pharmacists as self-care enablers given their <3% utilization across nearly all symptoms; culturally co-designed health literacy interventions to address the confidence–

	behaviour mismatch; redesign of NHS digital resources given <7% utilisation; and cross-sectoral action on structural barriers including subsidised OTC schemes for lower-income groups. Also, calling for a national symptom management strategy has been added.

VERSION 2 - REVIEW

**Reviewer**	**3**
**Name**	**Moinuddin, Mohammed**
**Affiliation**	**University of Lancashire, School of Medicine and Dentistry**
**Date**	**10-Aug-2026**
**COI**	**None**

An interesting descriptive study. Further attention is required around the statistical analysis, their justification and clarification. Please see my comments as follows:

Comment 1: (page6; lines 13-25) The manuscript reported that “care networks and social media data collected were stored on the Imperial College London secure database." I wonder which social media was used for data collection. Was the questionnaire publicly posted on social platforms or sent personally to their inbox? The questionnaire contained 83 questions, which is huge, and people tend to skip or click randomly on options when they see a long questionnaire. Would the author clarify further on these?

In addition, the study used a convenience online sample recruited through social media and professional networks rather than probability sampling. Consequently, the study population is unlikely to represent the UK adult population. Please clarify that the analyses estimate associations only within this convenience sample and avoid implying national representativeness. If demographic benchmarks are available, consider applying post-stratification or raking weights, or justify why weighting was not considered.

Comment 2: (page 5; lines 3-11) The author mentioned that previous studies have already covered a big area of this research. However, I can not specify the significant objective or novelty of this research. Would you please properly explain the research gap and the novelty of this research briefly?

Comment 3: (Measured and Variables) As this research used a 5-point ordinal Likert scale to assess individual self-care, confidence, and knowledge, it would be great to see Cronbach's alpha reliability statistics. Also, please ensure the questionnaire items were referenced.

Comment 4: Ordinal logistic regression was used for all primary analyses. However, no assessment of the proportional odds assumption is reported. Because proportional odds is

the key assumption underlying ordinal logistic regression, please report how this assumption was assessed.

In addition, the adjusted odds ratios are reported without assessing the overall model performance. Please report model diagnostic elements to allow readers to evaluate model adequacy.

Comment 5: Although multiple demographic variables were included in the regression models, there is no assessment of multicollinearity among predictors. Please report multicollinearity diagnostics (variance inflation factors, or tolerance values) and explain how potential collinearity was addressed. Moreover, the manuscript states that adjusted models controlled for several demographic variables. However, the rationale for selecting confounders is not clearly described. Please clarify whether variables were selected based on prior knowledge, literature, or statistical criteria. Avoid selecting adjustment variables solely on statistical significance.

VERSION 2 - AUTHOR RESPONSE

Reviewer 3	
An interesting descriptive study. Further attention is required around the statistical analysis, their justification and clarification. Please see my comments as follows:	We thank Reviewer 3 for this positive appraisal and for the constructive focus on statistical justification and clarification, which has strengthened the paper. Detailed point-by-point responses to each comment are provided below.
Comment 1: (page6; lines 13-25) The manuscript reported that “care networks and social media data collected were stored on the Imperial College London secure database." I wonder which social media was used for data collection. Was the questionnaire publicly posted on social platforms or sent personally to their inbox? The questionnaire contained 83 questions, which is huge, and people tend to skip or click randomly on options when they see a long questionnaire. Would the author clarify further on these?<br>In addition, the study used a convenience online sample recruited through social media and professional networks rather than probability sampling. Consequently, the study population is unlikely to represent the UK adult population. Please clarify that the analyses estimate associations only within this convenience	**Recruitment:**<br>The survey link was posted publicly on the social media accounts of the Self-Care Academic Research Unit and the Self-Care Forum (X and LinkedIn) and circulated through partner organisations' mailing lists and professional networks. It was open to anyone who saw it; nothing was sent to individual inboxes and no personal contact details were held. We note that the sentence the reviewer quotes had two sentences run together, which obscured the recruitment route; this has been corrected in the Methods.<br><br>**Survey length:**<br>Adaptive questioning meant no respondent was shown all 83 items, as items were displayed conditionally on prior responses. We have also examined

sample and avoid implying national representativeness. If demographic benchmarks are available, consider applying post-stratification or raking weights, or justify why weighting was not considered.	satisficing directly by quantifying straightlining within the two longest Likert blocks; the rates were low and give no indication of the patterned or random responding the reviewer anticipates. Both points are now stated in the Methods, with the figures reported there.<br><br>**Representativeness:**<br>We accept this fully and have revised the framing throughout, removing any implication of national representativeness and stating explicitly that the analyses estimate associations within this convenience sample. The main departures from the UK adult population are now quantified in the Limitations so that readers can judge their direction, and a corresponding bullet has been added to the Strengths and Limitations box.<br><br>On weighting, we considered post-stratification and raking and concluded neither would be appropriate here. Our estimands are conditional associations rather than population prevalences; the demographic variables one would weight on are already covariates in every adjusted model, so compositional differences are conditioned out; and weighting to observed margins cannot correct selection on the characteristics that plausibly drove participation in an open survey about self-care, such as topic interest and digital access, which are unmeasured and likely related to the outcomes. Weighting would add variance without addressing the principal source of bias, and risks conveying a representativeness the design cannot support. This reasoning is now set out in the Methods.
Comment 2: (page 5; lines 3-11) The author mentioned that previous studies have already covered a big area of this research. However, I can not specify the	We accept that the research gap was not stated sharply enough and have rewritten the closing paragraph of the Introduction. The companion paper analysed the

significant objective or novelty of this research. Would you please properly explain the research gap and the novelty of this research briefly?	public–professional interface and did not model the general public's own self-care engagement. The present paper addresses three questions it left open: which demographic characteristics independently predict self-care confidence and health information-seeking once other characteristics are held constant; which barriers the public itself identifies, as distinct from those attributed to it by professionals; and how symptom management and use of professional resources vary across specific common conditions. The third element allows the scale of contemporary self-management to be set against the last major UK characterisation of the symptom iceberg over a decade earlier and the magnitude of that shift is reported in the revised Introduction and Discussion.
Comment 3: (Measured and Variables) As this research used a 5-point ordinal Likert scale to assess individual self-care, confidence, and knowledge, it would be great to see Cronbach's alpha reliability statistics. Also, please ensure the questionnaire items were referenced.	The self-care confidence items did not use a five-point scale. They used a four-point forced-choice scale with no neutral midpoint; our Methods incorrectly described them as five-point and listed a response option that was never presented to respondents. This has been corrected, and the attitudinal, information-seeking and self-care activity items are now described accurately and separately.<br><br>We have added internal consistency statistics for the three confidence items and for the health literacy block, reported in the Methods. Because the items are ordinal, we report ordinal alpha from the polychoric correlation matrix alongside Cronbach's alpha. Both indicate acceptable to good internal consistency.<br><br>We would ask the reviewer to note one qualification, now stated in the paper: the confidence items were analysed as separate single-item outcomes rather than summed into a composite, so

	internal consistency is not a precondition for the analyses reported. These statistics are presented as evidence that the items behave coherently rather than as psychometric validation, and we retain our caveat that they constitute an unvalidated self-appraisal.<br><br>On provenance, the health literacy items were adapted from the European Health Literacy Survey Questionnaire and the remaining items drawn from the Self-Care Forum's Living Self-Care Survey instrument, now cited in the Methods. The full instrument is reproduced in the supplementary material of the companion paper reporting the same survey, which is cited in the Methods.
Comment 4: Ordinal logistic regression was used for all primary analyses. However, no assessment of the proportional odds assumption is reported. Because proportional odds is the key assumption underlying ordinal logistic regression, please report how this assumption was assessed.<br>In addition, the adjusted odds ratios are reported without assessing the overall model performance. Please report model diagnostic elements to allow readers to evaluate model adequacy.	We have assessed the parallel regression assumption using the Brant test, evaluated globally and per covariate, and added model adequacy diagnostics for all three models. Both are now reported in the Methods and in a new Results sub-section.<br><br>Two features of the data required a workaround. The lowest outcome categories were sparsely populated, and the lowest education category contained only seven respondents; together these produced quasi-complete separation in the lowest binary contrast, so the test was not estimable on the full specifications. We combined these sparse categories for the purposes of the assumption test only, which yields estimates differing from those reported by less than 0.05 on the odds ratio scale.<br><br>The global test indicated departure from proportionality in all three models. Rather than treat this as a global failure we examined each departure covariate by covariate. Five covariates were flagged in the self-care knowledge model, three in the healthy lifestyle model and five in

	the information-seeking model; all remaining covariates were consistent with proportional odds. For each flagged covariate that was also a significant predictor, threshold-specific estimates preserved the direction and significance of the pooled estimate in all but two cases. The first is Black British ethnicity in the self-care knowledge model, whose association is concentrated at the highest confidence category; we regard this as a substantive finding and now report it in the Results and Discussion. The second is Northern Ireland residency in the healthy lifestyle model, estimated on 69 respondents, which we have added to the existing limitation on underpowered subgroup analyses.<br><br>We have therefore retained the ordinal specification, which remains appropriate for the covariates carrying our substantive conclusions, with explicit caveats on the two affected estimates. We would note that with 27 covariates and an analytic sample of 3,011 the omnibus Brant test is highly powered and will detect departures too small to alter any inference.<br><br>On model performance, all three models fitted significantly better than the intercept-only model, and we now report likelihood ratio tests, pseudo-R^2 measures, AIC and BIC for each. These models were specified to estimate adjusted associations rather than to predict individual behaviour, so the pseudo-R^2 values indicate that demographic position is one of several determinants of self-care engagement, not that the estimated associations are imprecise. The precision of those estimates is reflected in the confidence intervals reported throughout.
Comment 5: Although multiple demographic variables were included in	**Multicollinearity:**

the regression models, there is no assessment of multicollinearity among predictors. Please report multicollinearity diagnostics (variance inflation factors, or tolerance values) and explain how potential collinearity was addressed. Moreover, the manuscript states that adjusted models controlled for several demographic variables. However, the rationale for selecting confounders is not clearly described. Please clarify whether variables were selected based on prior knowledge, literature, or statistical criteria. Avoid selecting adjustment variables solely on statistical significance.	Formal diagnostics are now reported in the Methods. Because several predictors are multi-category, we report generalised variance inflation factors alongside indicator-level VIF and tolerance, since indicator-level VIF is not invariant to the choice of reference category where a variable is represented by more than one dummy. All GVIF^(1/2×df) values lay between 1.01 and 1.13, indicating no meaningful collinearity between predictor sets, and no predictor was removed or combined on collinearity grounds.<br>We draw the reviewer's attention to one diagnostic that requires explanation. Indicator-level variance inflation factors for the education dummies are high (48.8 to 109.5). This is not evidence of collinearity between education and the other predictors; it is an arithmetic consequence of using primary school as the reference category, which contains only seven respondents. The generalised variance inflation factor for education, which does not depend on the choice of reference category, is 1.02 — among the lowest of any predictor set in the model. All other indicator-level factors were ≤3.48, with a minimum tolerance of 0.29. We have therefore retained the original coding so that the education categories remain as pre-specified, and have strengthened the existing caution in the Limitations that the education estimates rest on a very small reference category, yield unstable estimates with wide confidence intervals, and should not be over-interpreted.<br>**Confounder selection:**<br>We confirm that no statistical criterion was used at any stage. All covariates were specified a priori on conceptual and literature grounds and the same fully adjusted model was fitted for every outcome. No stepwise, forward or backward selection was applied, and no

	variable was retained or dropped based on its p-value. An explicit statement to this effect has been added to the Methods.

VERSION 3 - REVIEW

**Reviewer**	**3**
**Name**	**Moinuddin, Mohammed**
**Affiliation**	**University of Lancashire, School of Medicine and Dentistry**
**Date**	**31-Aug-2026**
**COI**	

We thank the authors for their substantial clarification and improvement of the statistical methodology and reporting. The manuscript is now more robust, reproducible, and transparent. All the required changes are incorporated in the main body of text as well.
