# Supplementary material for: What are the demographic predictors and barriers to self-care engagement in the UK? a cross-sectional survey study
Source: BMJ Open. 2026 Sep 21;16(9):e110380. doi: 10.1136/bmjopen-2025-110380 (PMC13599892; doi:10.1136/bmjopen-2025-110380)
Supplement: Supplementary file 2 [file bmjopen-16-9-s001.docx]

**Supplementary Table 1**

**Demographic characteristics of the study sample**

| **Variable (N=3,255)** | **Frequency (%)** |
| --- | --- |
| **Age** |  |
| 18-24 | 318 (9.8%) |
| 25-34 | 748 (23.0%) |
| 35-44 | 732 (22.5%) |
| 45-54 | 556 (17.1%) |
| 55-64 | 594 (18.3%) |
| 65+ | 307 (9.4%) |
| **Gender** |  |
| Female | 1,695 (52.1%) |
| Male | 1,526 (46.9%) |
| Other | 34 (1.0%) |
| **Ethnic group or background** |  |
| White | 2,682 (82.4%) |
| Asian, or Asian British | 213 (6.5%) |
| Black, Black British, Caribbean or African | 154 (4.7%) |
| Mixed or Multiple ethnic groups | 124 (3.8%) |
| Other ethnic groups | 55 (1.7%) |
| Prefer not to say | 27 (0.8%) |
| **Residency** |  |
| England | 2,795 (85.9%) |
| Scotland | 255 (7.8%) |
| Wales | 136 (4.2%) |
| Northern Ireland | 69 (2.1%) |
| **Highest level of education** |  |
| Primary school | 7 (0.2%) |
| Secondary school | 401 (12.3%) |
| Higher or secondary or further education (A-levels, BTEC, etc.) | 693 (21.3%) |
| Postgraduate training leading to qualification, diploma or equivalent | 658 (20.2%) |
| College or university degree | 1,483 (45.6%) |
| Prefer not to say | 13 (0.4%) |
| **Current employment status** |  |
| Employed full-time | 1,594 (49.0%) |
| Employed part-time | 472 (14.5%) |
| Retired | 371 (11.4%) |
| Self-employed full-time | 149 (4.6%) |
| Self-employed part-time | 150 (4.6%) |
| Student | 158 (4.9%) |
| Unemployed | 310 (9.5%) |
| Prefer not to say | 51 (1.6%) |
| **Disability** |  |
| No | 2,769 (85.1%) |
| Yes | 416 (12.8%) |
| Prefer not to say | 70 (2.2%) |
