# Supplementary material for: What are the demographic predictors and barriers to self-care engagement in the UK? a cross-sectional survey study
Source: BMJ Open. 2026 Sep 21;16(9):e110380. doi: 10.1136/bmjopen-2025-110380 (PMC13599892; doi:10.1136/bmjopen-2025-110380)
Supplement: Supplementary file 3 [file bmjopen-16-9-s002.docx]

**Supplementary Table 2**

**Managing Common Conditions**

| **Variable (N=3,255)** | **Frequency (%)** |
| --- | --- |
| **Have you had one of the following in the past 2 weeks?** |  |
| Headaches | 1,610 (49.5%) |
| Back pain | 1,077 (33.1%) |
| Joint Pain | 881 (27.1%) |
| Sore throat | 427 (13.1%) |
| Indigestion/heartburn | 584 (17.9%) |
| Cough | 455 (14.0%) |
| Cold or flu symptoms | 412 (12.7%) |
| Covid | 53 (1.6%) |
| Diarrhoea | 397 (12.2%) |
| Constipation | 319 (9.8%) |
| **Headaches (N = 1,610)** |  |
| Did nothing/waited | 403 (25.0%) |
| Looked for information on the internet | 72 (4.5%) |
| Discussed with family & friends | 88 (5.5%) |
| Checked the NHS site | 35 (2.2%) |
| I called 111 | 8 (0.5%) |
| Took over the counter medicines | 1,157 (71.9%) |
| Consulted a nurse | 8 (0.5%) |
| Consulted a pharmacist | 11 (0.7%) |
| Consulted a GP | 13 (0.8%) |
| Consulted complementary therapist | 4 (0.3%) |
| Took a prescribed medicine | 61 (3.8%) |
| **Back pain (N = 1,077)** |  |
| Did nothing/waited | 460 (42.7%) |
| Looked for information on the internet | 106 (9.8%) |
| Discussed with family & friends | 98 (9.1%) |
| Checked the NHS site | 45 (4.2%) |
| I called 111 | 4 (0.4%) |
| Took over the counter medicines | 441 (41.0%) |
| Consulted a nurse | 12 (1.1%) |
| Consulted a pharmacist | 11 (1.0%) |
| Consulted a GP | 36 (3.3%) |
| Consulted complementary therapist | 24 (2.0%) |
| Took a prescribed medicine | 85 (7.9%) |
| **Sore throat (N = 427)** |  |
| Did nothing/waited | 186 (43.6%) |
| Looked for information on the internet | 25 (5.9%) |
| Discussed with family & friends | 25 (5.9%) |
| Checked the NHS site | 12 (2.8%) |
| I called 111 | 5 (1.2%) |
| Took over the counter medicines | 215 (50.4%) |
| Consulted a nurse | 4 (0.9%) |
| Consulted a pharmacist | 10 (2.3%) |
| Consulted a GP | 8 (1.9%) |
| Consulted complementary therapist | 0 (0%) |
| Took a prescribed medicine | 9 (2.1%) |
| **Indigestion / Heartburn (N = 584)** |  |
| Did nothing/waited | 148 (25.3%) |
| Looked for information on the internet | 40 (6.9%) |
| Discussed with family & friends | 28 (4.8%) |
| Checked the NHS site | 12 (2.1%) |
| I called 111 | 2 (0.3%) |
| Took over the counter medicines | 343 (58.7%) |
| Consulted a nurse | 2 (0.3%) |
| Consulted a pharmacist | 10 (1.7%) |
| Consulted a GP | 11 (1.9%) |
| Consulted complementary therapist | 4 (0.7%) |
| Took a prescribed medicine | 77 (13.2%) |
| **Cough (N = 455)** |  |
| Did nothing/waited | 228 (50.1%) |
| Looked for information on the internet | 30 (6.6%) |
| Discussed with family & friends | 30 (6.6%) |
| Checked the NHS site | 14 (3.1%) |
| I called 111 | 1 (0.2%) |
| Took over the counter medicines | 168 (36.9%) |
| Consulted a nurse | 9 (2.0%) |
| Consulted a pharmacist | 13 (2.9%) |
| Consulted a GP | 17 (3.7%) |
| Consulted complementary therapist | 0 (0%) |
| Took a prescribed medicine | 18 (4.0%) |
| **Cold or flu symptoms (N = 412)** |  |
| Did nothing/waited | 153 (37.1%) |
| Looked for information on the internet | 20 (4.9%) |
| Discussed with family & friends | 32 (7.7%) |
| Checked the NHS site | 16 (3.9%) |
| I called 111 | 1 (0.2%) |
| Took over the counter medicines | 233 (56.6%) |
| Consulted a nurse | 7 (1.7%) |
| Consulted a pharmacist | 10 (2.4%) |
| Consulted a GP | 10 (2.4%) |
| Consulted complementary therapist | 0 (0%) |
| Took a prescribed medicine | 11 (2.7%) |
| **Joint pain (N = 881)** |  |
| Did nothing/waited | 467 (53.0%) |
| Looked for information on the internet | 40 (4.5%) |
| Discussed with family & friends | 35 (4.0%) |
| Checked the NHS site | 25 (2.8%) |
| I called 111 | 6 (0.7%) |
| Took over the counter medicines | 373 (42.3%) |
| Consulted a nurse | 3 (0.3%) |
| Consulted a pharmacist | 5 (0.6%) |
| Consulted a GP | 12 (1.4%) |
| Consulted complementary therapist | 2 (0.2%) |
| Took a prescribed medicine | 19 (2.2%) |
| **Covid (N = 53)** |  |
| Did nothing/waited | 10 (18.9%) |
| Looked for information on the internet | 8 (15.1%) |
| Discussed with family & friends | 9 (17.0%) |
| Checked the NHS site | 13 (24.5%) |
| I called 111 | 1 (1.9%) |
| Took over the counter medicines | 30 (56.6%) |
| Consulted a nurse | 2 (3.8%) |
| Consulted a pharmacist | 1 (1.9%) |
| Consulted a GP | 4 (7.6%) |
| Consulted complementary therapist | 1 (1.9%) |
| Took a prescribed medicine | 4 (7.6%) |
| **Diarrhoea (N = 397)** |  |
| Did nothing/waited | 230 (57.9%) |
| Looked for information on the internet | 26 (6.6%) |
| Discussed with family & friends | 12 (3.0%) |
| Checked the NHS site | 13 (3.3%) |
| I called 111 | 5 (1.3%) |
| Took over the counter medicines | 116 (29.2%) |
| Consulted a nurse | 5 (1.3%) |
| Consulted a pharmacist | 3 (0.8%) |
| Consulted a GP | 13 (3.3%) |
| Consulted complementary therapist | 1 (0.3%) |
| Took a prescribed medicine | 18 (4.5%) |
| **Constipation (N = 319)** |  |
| Did nothing/waited | 163 (51.1%) |
| Looked for information on the internet | 43 (13.5%) |
| Discussed with family & friends | 28 (8.8%) |
| Checked the NHS site | 10 (3.1%) |
| I called 111 | 1 (0.3%) |
| Took over the counter medicines | 110 (34.5%) |
| Consulted a nurse | 0 (0%) |
| Consulted a pharmacist | 7 (2.2%) |
| Consulted a GP | 8 (2.5%) |
| Consulted complementary therapist | 0 (0%) |
| Took a prescribed medicine | 17 (5.3%) |
