# Supplementary material for: What are the demographic predictors and barriers to self-care engagement in the UK? a cross-sectional survey study
Source: BMJ Open. 2026 Sep 21;16(9):e110380. doi: 10.1136/bmjopen-2025-110380 (PMC13599892; doi:10.1136/bmjopen-2025-110380)
Supplement: Supplementary file 4 [file bmjopen-16-9-s003.docx]

**Supplementary Table 3**

**Ordinal logistic regression analysis of demographic factors predicting knowledge and understanding of self-care practice**

| **Variable** | **Knowledge & understanding of self-care practice** | | | |
| --- | --- | --- | --- | --- |
|  | **Unadjusted** | | **Adjusted** | |
|  | **OR (95% CI)** | **P-value** | **aOR (95% CI)** | **P-value** |
| **Age** | | | |  |
| 18-24 | Ref |  | Ref |  |
| 25-34 | 1.14 (0.87 - 1.47) | 0.343 | 1.20 (0.88 - 1.66) | 0.253 |
| 35-44 | 1.25 (0.96 - 1.62) | 0.099 | 1.32 (0.96 - 1.82) | 0.093 |
| 45-54 | 1.15 (0.87 - 1.51) | 0.331 | 1.35 (0.97 - 1.89) | 0.077 |
| 55-64 | 1.79 (1.36 - 2.35) | **<0.001** | 2.05 (1.45 - 2.89) | **<0.001** |
| 65 and above | 2.62 (1.92 - 3.57) | **<0.001** | 3.22 (2.06 - 5.03) | **<0.001** |
| **Gender** | | | |  |
| Female | Ref |  | Ref |  |
| Male | 0.78 (0.68 - 0.89) | **<0.001** | 0.79 (0.68 - 0.91) | **0.002** |
| Other | 0.92 (0.47 - 1.78) | 0.800 | 1.09 (0.55 - 2.18) | 0.800 |
| **Ethnicity** | | | |  |
| White | Ref |  | Ref |  |
| Black / African Caribbean / Black British | 1.78 (1.28 - 2.47) | **0.001** | 1.99 (1.39 - 2.84) | **<0.001** |
| Asian / Asian British | 0.65 (0.49 - 0.85) | **0.002** | 0.66 (0.49 - 0.89) | **0.006** |
| Mixed or Multiple | 0.88 (0.61 - 1.25) | 0.467 | 1.03 (0.71 - 1.51) | 0.860 |
| Other ethnic group | 0.86 (0.51 - 1.44) | 0.558 | 0.83 (0.48 - 1.45) | 0.519 |
| **Highest level of education** | | | |  |
| Primary school | Ref |  | Ref |  |
| Secondary school | 0.44 (0.01 - 1.94) | 0.276 | 0.32 (0.07 - 1.46) | 0.141 |
| Higher or secondary or further education (A-levels, BTEC, etc) | 0.37 (0.08 - 1.62) | 0.185 | 0.30 (0.07 - 1.36) | 0.117 |
| Postgraduate training leading to qualification | 0.57 (0.13 - 2.51) | 0.458 | 0.47 (0.10 - 2.16) | 0.332 |
| College or university degree | 0.46 (0.11 - 2.01) | 0.307 | 0.39 (0.09 - 1.76) | 0.220 |
| **Employment status** |  |  |  |  |
| Unemployed | Ref |  | Ref |  |
| Student | 1.03 (0.70 - 1.50) | 0.895 | 1.06 (0.67 - 1.68) | 0.791 |
| Self-employed part-time | 1.49 (1.02 - 2.18) | **0.041** | 1.12 (0.74 - 1.68) | 0.595 |
| Self-employed full-time | 1.54 (1.05 - 2.25) | **0.028** | 1.23 (0.82 - 1.86) | 0.315 |
| Retired | 2.12 (1.57 - 2.85) | **<0.001** | 1.08 (0.73 - 1.60) | 0.689 |
| Employed part-time | 1.40 (1.05 - 1.85) | **0.021** | 1.01 (0.74 - 1.37) | 0.957 |
| Employed full-time | 1.18 (0.93 - 1.51) | 0.168 | 0.96 (0.73 - 1.25) | 0.742 |
| **Disability** | | | |  |
| No | Ref |  | Ref |  |
| Yes | 0.65 (0.53 - 0.80) | **<0.001** | 0.69 (0.55 - 0.88) | **0.002** |
| **Long-term condition** |  |  |  |  |
| No | Ref |  | Ref |  |
| Yes | 0.85 (0.74 - 0.97) | **0.021** | 0.76 (0.64 - 0.90) | **0.001** |
| **Healthcare professional** | | | |  |
| No | Ref |  | Ref |  |
| Yes | 1.55 (1.18 - 2.02) | **0.001** | 1.51 (1.13 - 2.02) | **0.006** |
| **Residency** |  |  |  |  |
| England | Ref |  | Ref |  |
| Wales | 0.96 (0.68 - 1.35) | 0.808 | 0.98 (0.69 - 1.40) | 0.911 |
| Scotland | 0.81 (0.63 - 1.04) | 0.097 | 0.78 (0.60 - 1.02) | 0.068 |
| Northern Ireland | 0.53 (0.34 - 0.85) | **0.008** | 0.55 (0.34 - 0.91) | **0.020** |
| (Outcome: Not at all confident, Not very confident, Fairly confident or Very confident). *Adjusted for age, gender, ethnicity, highest level of education, employment status, disability, long-term conditions, being a healthcare professional or not and residency.* | | | | |
