# Supplementary material for: What are the demographic predictors and barriers to self-care engagement in the UK? a cross-sectional survey study
Source: BMJ Open. 2026 Sep 21;16(9):e110380. doi: 10.1136/bmjopen-2025-110380 (PMC13599892; doi:10.1136/bmjopen-2025-110380)
Supplement: Supplementary file 5 [file bmjopen-16-9-s004.docx]

**Supplementary Table 4**

**Ordinal logistic regression analysis of demographic factors predicting confidence in the knowledge and understanding of leading a healthy lifestyle**

| **Variable** | **Knowledge & understanding to lead a healthy lifestyle** | | | |
| --- | --- | --- | --- | --- |
|  | **Unadjusted** | | **Adjusted** | |
|  | **OR (95% CI)** | **P-value** | **aOR (95% CI)** | **P-value** |
| **Age** | | | |  |
| 18-24 | Ref |  | Ref |  |
| 25-34 | 1.03 (0.79 - 1.36) | 0.808 | 1.04 (0.75 - 1.44) | 0.822 |
| 35-44 | 1.34 (1.02 - 1.75) | 0.035 | 1.34 (0.96 - 1.87) | 0.081 |
| 45-54 | 1.29 (0.98 - 1.72) | 0.074 | 1.51 (1.07 - 2.13) | **0.020** |
| 55-64 | 1.92 (1.45 - 2.54) | **<0.001** | 2.11 (1.48 - 3.00) | **<0.001** |
| 65 and above | 2.63 (1.92 - 3.62) | **<0.001** | 2.96 (1.87 - 4.68) | **<0.001** |
| **Gender** | | | |  |
| Female | Ref |  | Ref |  |
| Male | 1.04 (0.91 - 1.20) | 0.57 | 1.10 (0.94 - 1.28) | 0.231 |
| Other | 1.21 (0.62 - 2.34) | 0.56 | 1.39 (0.69 - 2.76) | 0.355 |
| **Ethnicity** | | | |  |
| White | Ref |  | Ref |  |
| Black / African Caribbean / Black British | 1.21 (0.87 - 1.67) | 0.255 | 1.21 (0.85 - 1.73) | 0.291 |
| Asian / Asian British | 0.49 (0.36 - 0.64) | **<0.001** | 0.46 (0.34 - 0.63) | **<0.001** |
| Mixed or Multiple | 0.98 (0.68 - 1.42) | 0.927 | 1.12 (0.75 - 1.65) | 0.583 |
| Other ethnic group | 0.92 (0.53 - 1.60) | 0.778 | 0.95 (0.53 - 1.70) | 0.858 |
| **Highest level of education** | | | |  |
| Primary school | Ref |  | Ref |  |
| Secondary school | 0.34 (0.08 - 1.42) | 0.138 | 0.21 (0.05 - 0.89) | **0.034** |
| Higher or secondary or further education (A-levels, BTEC, etc) | 0.34 (0.08 - 1.42) | 0.139 | 0.24 (0.06 - 1.03) | 0.055 |
| Postgraduate training leading to qualification | 0.53 (0.13 - 2.19) | 0.380 | 0.41 (0.10 - 1.74) | 0.225 |
| College or university degree | 0.44 (0.11 - 1.80) | 0.251 | 0.33 (0.08 - 1.38) | 0.128 |
| **Employment status** |  |  |  |  |
| Unemployed | Ref |  | Ref |  |
| Student | 1.11 (0.75 - 1.65) | 0.603 | 1.21 (0.76 - 1.95) | 0.424 |
| Self-employed part-time | 1.69 (1.14 - 2.52) | **0.009** | 1.22 (0.80 - 1.86) | 0.357 |
| Self-employed full-time | 1.86 (1.26 - 2.75) | **0.002** | 1.29 (0.85 - 1.97) | 0.237 |
| Retired | 2.48 (1.83 - 3.37) | **<0.001** | 1.25 (0.84 - 1.86) | 0.277 |
| Employed part-time | 1.51 (1.13 - 2.03) | **0.006** | 1.07 (0.78 - 1.47) | 0.654 |
| Employed full-time | 1.34 (1.04 - 1.73) | **0.022** | 0.96 (0.73 - 1.27) | 0.786 |
| **Disability** | | | |  |
| No | Ref |  | Ref |  |
| Yes | 0.52 (0.42 - 0.65) | **<0.001** | 0.58 (0.45 - 0.74) | **<0.001** |
| **Long-term condition** |  |  |  |  |
| No | Ref |  | Ref |  |
| Yes | 0.73 (0.63 - 0.84) | **<0.001** | 0.65 (0.55 - 0.77) | **<0.001** |
| **Healthcare professional** | | | |  |
| No | Ref |  | Ref |  |
| Yes | 1.55 (1.19 - 2.03) | **0.001** | 1.66 (1.24 - 2.23) | **0.001** |
| **Residency** |  |  |  |  |
| England | Ref |  | Ref |  |
| Wales | 0.87 (0.62 - 1.23) | 0.436 | 0.90 (0.63 - 1.30) | 0.580 |
| Scotland | 0.86 (0.66 - 1.11) | 0.243 | 0.79 (0.60 - 1.04) | 0.087 |
| Northern Ireland | 0.51 (0.32 - 0.81) | **0.005** | 0.55 (0.33 - 0.90) | **0.017** |
| Outcome: Not at all confident, Not very confident, Fairly confident or Very confident. *Adjusted for age, gender, ethnicity, highest level of education, employment status, disability, long-term conditions, being a healthcare professional or not and residency.* | | | | |
