# Supplementary material for: What are the demographic predictors and barriers to self-care engagement in the UK? a cross-sectional survey study
Source: BMJ Open. 2026 Sep 21;16(9):e110380. doi: 10.1136/bmjopen-2025-110380 (PMC13599892; doi:10.1136/bmjopen-2025-110380)
Supplement: Supplementary file 6 [file bmjopen-16-9-s005.docx]

**Supplementary Table 5**

**Ordinal logistic regression analysis of demographic factors predicting the ability to find relevant health information when faced with a common condition**

| **Variable** | **Finding health information when having a common condition** | | | |
| --- | --- | --- | --- | --- |
|  | **Unadjusted** | | **Adjusted** | |
|  | **OR (95% CI)** | **P-value** | **aOR (95% CI)** | **P-value** |
| **Age** | | | |  |
| 18-24 | Ref |  | Ref |  |
| 25-34 | 1.26 (0.96 - 1.66) | 0.089 | 1.17 (0.85 - 1.62) | 0.345 |
| 35-44 | 1.80 (1.38 - 2.36) | **<0.001** | 1.62 (1.17 - 2.25) | **0.004** |
| 45-54 | 1.94 (1.46 - 2.57) | **<0.001** | 1.73 (1.23 - 2.43) | **0.002** |
| 55-64 | 2.20 (1.67 - 2.91) | **<0.001** | 1.93 (1.36 - 2.73) | **<0.001** |
| 65 and above | 2.00 (1.46 - 2.75) | **<0.001** | 1.78 (1.13 - 2.80) | **0.012** |
| **Gender** | | | |  |
| Female | Ref |  | Ref |  |
| Male | 0.75 (0.65 - 0.86) | **<0.001** | 0.73 (0.63 - 0.85) | **<0.001** |
| Other | 1.54 (0.78 - 3.04) | 0.212 | 1.68 (0.83 - 3.37) | 0.147 |
| **Ethnicity** | | | |  |
| White | Ref |  | Ref |  |
| Black / African Caribbean / Black British | 0.48 (0.34 - 0.67) | **<0.001** | 0.56 (0.39 - 0.81) | **0.002** |
| Asian / Asian British | 0.65 (0.49 - 0.87) | **0.003** | 0.70 (0.52 - 0.98) | **0.035** |
| Mixed or Multiple | 0.67 (0.47 - 0.96) | **0.031** | 0.82 (0.56 - 1.20) | 0.310 |
| Other ethnic group | 0.68 (0.40 - 1.17) | 0.163 | 0.83 (0.47 - 1.47) | 0.526 |
| **Highest level of education** | | | |  |
| Primary school | Ref |  | Ref |  |
| Secondary school | 4.34 (0.86 - 21.88) | 0.076 | 3.43 (0.66 - 17.78) | 0.076 |
| Higher or secondary or further education (A-levels, BTEC, etc) | 3.05 (0.61 - 15.33) | 0.175 | 2.60 (0.50 - 13.42) | 0.253 |
| Postgraduate training leading to qualification | 3.71 (0.74 - 18.63) | 0.111 | 3.19 (0.62 - 16.49) | 0.166 |
| College or university degree | 3.47 (0.69 - 17.35) | 0.130 | 3.12 (0.61 - 16.05) | 0.130 |
| **Employment status** |  |  |  |  |
| Unemployed | Ref |  | Ref |  |
| Student | 0.66 (0.45 - 0.96) | **0.031** | 1.00 (0.63 - 1.58) | 0.998 |
| Self-employed part-time | 1.28 (0.87 - 1.88) | 0.209 | 1.04 (0.69 - 1.57) | 0.859 |
| Self-employed full-time | 1.08 (0.73 - 1.59) | 0.708 | 0.95 (0.62 - 1.45) | 0.816 |
| Retired | 1.39 (1.03 - 1.87) | **0.031** | 1.10 (0.75 - 1.63) | 0.626 |
| Employed part-time | 0.98 (0.73 - 1.30) | 0.870 | 0.82 (0.60 - 1.11) | 0.200 |
| Employed full-time | 1.03 (0.81 - 1.31) | 0.818 | 1.03 (0.79 - 1.35) | 0.802 |
| **Disability** | | | |  |
| No | Ref |  | Ref |  |
| Yes | 1.20 (0.98 - 1.47) | 0.080 | 1.16 (0.92 - 1.47) | 0.215 |
| **Long-term condition** |  |  |  |  |
| No | Ref |  | Ref |  |
| Yes | 1.09 (0.95 - 1.25) | 0.238 | 0.88 (0.75 - 1.04) | 0.149 |
| **Health and care professional** | | | |  |
| No | Ref |  | Ref |  |
| Yes | 0.93 (0.71 - 1.22) | 0.600 | 1.09 (0.81 - 1.46) | 0.578 |
| **Residency** |  |  |  |  |
| England | Ref |  | Ref |  |
| Wales | 1.06 (0.76 - 1.48) | 0.736 | 0.97 (0.69 - 1.39) | 0.887 |
| Scotland | 0.94 (0.72 - 1.21) | 0.610 | 0.89 (0.68 - 1.16) | 0.393 |
| Northern Ireland | 0.71 (0.44 - 1.15) | 0.162 | 0.68 (0.41 - 1.13) | 0.139 |
| Outcome: Strongly disagree, Disagree, Neither agree nor disagree, Agree or Strongly agree) *Ordinal logistic regression analysis of demographic factors predicting When I have a common condition, I try to find health information for myself (Outcome: Strongly disagree, Disagree, Neither agree nor disagree, Agree or Strongly agree). Adjusted for age, gender, ethnicity, highest level of education, employment status, disability, long-term conditions, being a health and care professional or not and residency.* | | | | |
